# Behaviour of Tetrabenazine in Acid Medium: Reassessment and Impact on Formulation

**DOI:** 10.3390/pharmaceutics11010044

**Published:** 2019-01-20

**Authors:** Laurent Ettouati, Zoé Senta-Loys, Sandrine Bourgeois, Bernard Fenet, Marc Le Borgne, Hatem Fessi

**Affiliations:** 1 EA 4446 Bioactive Molecules and Medicinal Chemistry, SFR Santé Lyon-Est CNRS UMS3453-INSERM US7, Faculté de Pharmacie–ISPB, Université Claude Bernard Lyon, Université de Lyon, 18 avenue Rockefeller, F-69373, Lyon Cedex 8, France; marc.le-borgne@univ-lyon1.fr; 2 LAGEP UMR 5007, CNRS, Université Claude Bernard Lyon 1, Université de Lyon, 43 bd du 11 novembre, F-69622 Villeurbanne, France; zoe.senta@gmail.com (Z.S.-L.); sandrine.bourgeois@univ-lyon1.fr (S.B.); hatem.fessi@univ-lyon1.fr (H.F.); 3 ISPB-Faculté de Pharmacie, Université Claude Bernard-Lyon 1, Université de Lyon, 8 avenue Rockefeller, F-69373, Lyon Cedex 8, France; 4 Centre Commun de RMN, Université Lyon 1, Université de Lyon, 43 boulevard du 11 Novembre, F-69622 Villeurbanne Cedex, France; bernard.fenet@gmail.com

**Keywords:** tetrabenazine, deuteration, deuterated tetrabenazine, synthesis, stability, degradation impurities, acid medium, LC-MS, orodispersible film

## Abstract

Thorough studies of previous analytical stress data of tetrabenazine, a dopamine depleting agent, showed a potential susceptibility to acidic conditions. Hence, the behavior of tetrabenazine acidic solutions was studied by LC-MS and NMR spectroscopy. Reverse-phase LC-MS analysis of tetrabenazine acidic aqueous solutions consistently showed a main lipophilic impurity in a proportion of 15 to 20%. NMR spectroscopy studies did not allow to completely ascertain its structure. However, we hypothesize an interconversion of *trans*-tetrabenazine with its unstable *cis* isomer via an open isoquinolinium intermediate. Evaluation of tetrabenazine integrity in orodispersible films was reassessed in light of these observations after formulation and during stability study. Even if interconversion of *trans*-tetrabenazine with its *cis* isomer was observed in orodispersible films containing tetrabenazine, this phenomenon seems not to have any consequences for the overall tetrabenazine bioavailability.

## 1. Introduction

**Figure 1:**
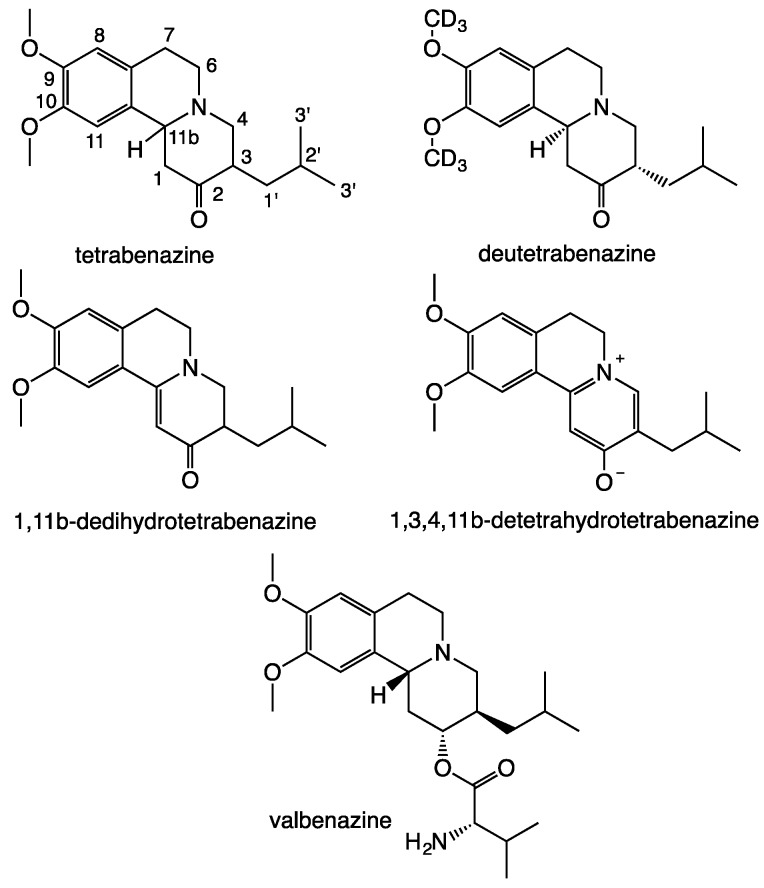
Structures of tetrabenazine (TBZ), deuterated TBZ, two tetrabenazine photolytic impurities and valbenazine.

Tetrabenazine (3-isobutyl-9,10-dimethoxy-1,3,4,6,7,11b-hexahydro-2*H*-pyrido[2,1-*a*]isoquinolin-2-one, TBZ, Xenazine^®^, Figure 1), a dopamine depleting agent, is clinically used to manage movement disorders like chorea associated with Huntington’s disease as well as off-label symptomatic treatment of many other hyperkinetic movement disorder [1,2,3]. More recently, valbenazine (Ingrezza^®^) [4,5,6], an isoleucine derivative of a tetrabenazine metabolite and deutetrabenazine (Austedo^®^), have been approved for treatment of same pathologies [7,8]. Deuterated drugs present as deutetrabenazine, the first approved deuterated drug, and have improved stability and better pharmacokinetics than their hydrogenated counterparts [9,10,11,12,13].

Previous stability study of TBZ solution under Suntest^®^ light exposure [14] has shown two major TBZ impurities identified as dedihydrotetrabenazine (DTBZ) and detetrahydrotetrabenazine (TTBZ), associated to strong discoloration (Figure 1). Close examination of reverse-phase LC-MS chromatograms revealed one additional weak peak at higher retention time in all TBZ samples exposed to acid stress conditions. Orodispersible films (ODF) of TBZ were previously developed for the paediatric administration [15,16]. This new form of drug administration called “ready to use” offers many advantages, such as no water needed for administration, enhanced pediatric population compliance (>6 years), possibility of local action, dose accuracy compared to syrup, control of manufacturing processes, ease of handling and ease of portablity [17,18]. ODF were obtained by “casting/solvent evaporation” method [19] with TBZ pre-solubilization in acidic aqueous solution (citric acid). However, yellowing of TBZ ODF was observed during stability studies. This discoloration has been correlated with results obtained by Bourezg et al. [14], showing yellowing of TBZ in aqueous solution after strong light exposure. Thus, evaluation of TBZ integrity in ODF was investigated directly after formulation and during a stability study (6 months, 40 °C, 75% HR) by LC-MS.

## 2. Materials and Methods 

### 2.1. Reagents and Solvents

TBZ was provided by Safic Alcan (Puteaux, France). All reagents and solvents were of analytical grade. Water used for the LC-MS analysis was prepared by using Purelab Option Q water purification system from ELGA LabWater (Antony, France).

### 2.2. Forced Acid Degradation Studies

Stock solution of TBZ was prepared either in acetonitrile or citric acid aqueous solution (20 mg·mL^−1^) and diluted with equivalent volumes of various acid solutions to obtain mixture at TBZ concentration of 10 mg·mL^−1^ (Table 1). Each solution was compared to appropriate control. All samples were prepared away from light to avoid potential photolytic degradation. Each forced degradation condition was tested in triplicate.

### 2.3. Instrumentation and Chromatographic Separation Conditions

#### 2.3.1. LC-MS Analyses

Liquid chromatography (LC) analysis was carried out by ultra-performance liquid chromatography (UPLC) Agilent 1290 Infinity system coupled to an Agilent 6120 Quadrupole mass spectrometer equipped with an electrospray ionization source (positive ESI mode) (Agilent Technologies, Santa Clara, CA, USA). The chromatographic separation was performed on a reversed phase column Zorbax Eclipse + C18 Agilent (50 mm × 2.1 mm; 1.8 µm). The LC method and mass spectrometry analysis parameters were the same as described previously [14].

### 2.4. Syntheses and Structural Characterization of [1,1-di-^2^H,3-^2^H]-Tetrabenazine([1,1-di-^2^H,3-^2^H]-TBZ)

#### 2.4.1. Materials and Methods

NMR spectra were recorded on a Bruker AV300 (300 MHz for ^1^H and 75 MHz for ^13^C), a Bruker AV400 (400 MHz for ^1^H and 133 MHz for ^13^C) and a Bruker AV500 (500 MHz for ^1^H and 125 MHz for ^13^C and 2D experiments) using D_2_O, DMSO-*d*_6_ or D_2_O and DMSO-*d*_6_ in mixture with acetic acid or citric acid as solvents. Off-resonance ROESY (Rotating frame nuclear Overhauser effect spectroscopy) experiments were carried out according to Bax and Davis [20] and Desvaux et al. [21]. Spectra were processed with NMR notebook 2.80 (NMRTEC, Ilkirch-Graffenstaden, France) and TopSpin 4.0.6 (Bruker BioSpin, Billerica, MA, USA). Chemical shifts (*δ*) are referred to that of the solvent.

#### 2.4.2. Synthesis of [1,1-di-^2^H,3-^2^H]-Tetrabenazine

A tetrabenazine sample (0.150 g, 0.473 mmol) was dissolved in 1.5 M citric acid/D_2_O solution (15 mL), and the solution was refluxed for 16 h under inert atmosphere. The solution was then neutralized with sodium carbonate to pH 11 and extracted with ethyl acetate (3 × 20 mL). The combined organic phases were washed with brine, dried over sodium sulfate and concentrated under reduced pressure. The title product was obtained in quantitative yield. [1,1-di-^2^H,3-^2^H]-tetrabenazine. TLC (silica) *R*_f_ 0,43cyclohexane/ethyl acetate: 7:3 (UV+ 254 nm). NMR ^1^H (300 MHz, DMSO-*d*_6_, *δ* ppm) 6.69 (2 H, s, 2 HPh), 3.71 (3 H, s, OCH_3_), 3.44 (s, 1 H, H-11b), 3.24 (1 H, *J* = 11.2 Hz, H-4), 3.12 (1 H, m, H-6), 2.92 (1 H, m, H-7), 2.67 (1 H, m, H-7), 2.48 (1 H, m, H-6), 2.27 (1 H, d, *J* = 12.1 Hz, H-4), 1.80–1.62 (2 H, m, –CH_2_–CH(CH_3_)_2_), 1.29 (1 H, m, CH(CH_3_)_2_), 0.98 (3 H, d, *J* = 6.1 Hz, CH_3_), 0.93 (3 H, d, *J* = 6.1 Hz, CH_3_).^13^C NMR (75 MHz, CDCl_3_, *δ* ppm) 195.50 (C-2), 156.64 (C-9), 151.49 (C-10), 148.06 (C-11b), 128.98 (C-7a), 120.84 (C-11a), 110.41 (C-8 or C-11), 108.28 (C-11 or C-8), 94.27 (C-1), 56.12 (CH_3_O), 56.10 (CH_3_O), 55.83 (C-4), 49.12 (C-6), 42.03 (C-3), 37.53 (C-1’), 28.52 (C-7), 25.57 (C-2’), 23.60 (CH_3_), 21.89 (CH_3_).

### 2.5. Characterization of TBZ Integrity in ODF

#### 2.5.1. Extraction of TBZ 

The composition of each ODF analyzed are shown in Table 2. ODF were cut and weighted to obtain a mass of 10 mg. Each sample was introduced in a glass hemolysis tube containing 2 mL of ACN and mixed during 1 min with a vortex at 3000 rpm. Then, the solution was collected and diluted with ACN to obtain a TBZ concentration of 0.5 mg·mL^−1^. After filtration through a 0.22 μm Nylon membrane (Millipore^®^), samples were analyzed by reverse-phase LC-MS in the same conditions used for forced acid degradation studies.

#### 2.5.2. Condition of Stability Study

ODF were stored in sealed aluminum strips and stability (ICH Q1-R2) was investigated at 40 °C; 75% HR (accelerated storage conditions) for 6 months. Film samples of each formulation were withdrawn at 1, 3, and 6 months for LC-MS analyses.

## 3. Results

### 3.1. Acid-Forced Degradation Studies and LC-MS Analyses

Tetrabenazine behavior in different acid media was implemented as shown in Table 1. These analyses confirm initial observations indicating apparition in all samples treated with acid of an additional signal in LC-MS corresponding to a more lipophilic compound (*t*_r_ at 2.3 to 2.5 min) in 15 to 20% proportion (see Table 1 and Figure S1 in Supplementary Materials). It is interesting to note its presence in chromatogram of TBZ sample dissolved in ACN/pH 6.5 deionized water mixture heated to 70 °C for 70 h. On the other hand, TBZ is stable in solution in acetonitrile and kept away from light at 4 °C for at least one week. In isocratic condition used for chromatographic analysis, TBZ control elutes at *t*_r_ 1.85 min with a protonated molecular ion [MH]^+^ at *m/z* 318.2 and the unknown more lipophilic compound with *t*_r_ 2.3 to 2.5 min displays the same m/z ratio than TBZ.

### 3.2. NMR Studies of TBZ in Acid Medium

TBZ instability in an acid medium has been confirmed in ^1^H NMR by dissolving a TBZ sample in a mixture of deuterated acetic acid/D_2_O or 1.5 M citric acid/D_2_O with continuous monitoring (see Figure S2 in Supplementary Materials). This instability was practically immediate and did not evolve significantly with time on a scale of a few hours. The ^1^H NMR spectrum showed significant modifications in the chemical shifts of some TBZ protons, particularly H-11b probably deshielded by nitrogen protonation with the appearance of signals between 1.5 and 2 ppm and between 4.2 and 4.5 ppm (see Figure S3A,C in Supplementary Materials). Furthermore, aromatic protons area showed apparition of signals accounting for at least two additional compounds (see Figure S3B in Supplementary Materials). In DMSO-*d*_6_, TBZ H-8, and H-11 aromatic protons appeared at around 6.70 ppm as two close singlets while in 1.5 M citric acid/D_2_O solution three couples of singlets were observed at 6.56/6.41 ppm, 6.53/6.46 ppm and 6.51/6.40 ppm, respectively. Complementary 2D NMR spectra (^1^H-^1^H COSY, ^1^H-^13^C HSQC, ^1^H-^13^C HMBC experiments) did not identify with certainty the nature of these compounds that appeared in aqueous acidic environments. However, it has been noted an exchange phenomenon in ROESY spectrum for the 4.45 ppm signal. It must be stressed that TBZ spectrum stayed identical for one night in 1.5 M acetic acid/DMSO-*d*_6_ or DMSO-*d*_6_ alone. However, we noted puzzling simplification of TBZ spectra after 48 h in acetic acid/D_2_O with loss of coupling for the 4.30 ppm signal. We hypothesize some proton-deuterium exchange phenomena, which was confirmed by refluxing TBZ sample in 1.5 M citric acid/D_2_O for 16 h. The ^1^H NMR spectrum of the resulting instable compound, [1,1-di-^2^H,3-^2^H]-TBZ, shows the disappearance of protons H-1a/H-1b and H-3 with concomitant simplification of H-11b and H-4 (see Figure S4 in Supplementary Materials). Its formation is easily explained by enolization of the C=O in 2-position and subsequent deuteration.

### 3.3. LC-MS Analyses of TBZ Contained in ODF

TBZ results obtained after ODF extraction are summarized in Table 3 (see Figure S6 in Supplementary Materials for complete set of data). At *T* = 0, the appearance of a more lipophilic peak was observed in a proportion from 14.2 to 17.4% by LC-MS. After 3 months of stability, the percentage of the second more lipophilic component was decreased in sample F2 at 4.4%. This decrease was also observed in F4 after 6 months of stability, while, the proportion was consistent during this time in samples F1 and F3.

## 4. Discussion

**Figure 2:**
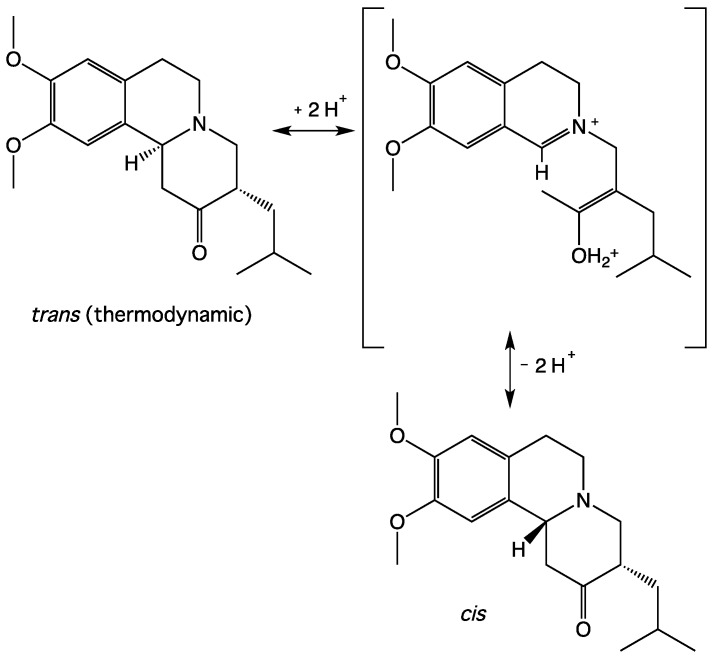
Hypothetical interconversion between *trans*-TBZ and *cis*-TBZ (only one enantiomer is depicted) in acidic medium.

TBZ is marketed as the racemic *trans* form due to thermodynamic instability of the *cis* isomers. The stereogenic instability of the 2-oxobenzo[a]quinolizine nucleus substituted in position 3 in acidic medium is well documented [22,23]. Thus, we hypothesize the interconversion of *trans*-TBZ via the open isoquinolinium structure (Figure 2) with *cis*-TBZ. This involves racemization at both centers C-11b and C-3 by reversible Mannich reaction (C-11b) and by reversible enolization (C-3) [23]. An exchange phenomenon in ROESY spectrum between signals at 4.45 ppm and 4.65 ppm (see Figure S5A in Supplementary Materials) has been noted which is possibly assigned to intermediate species (see Figure S5B in Supplementary Materials). In the case of acid aqueous stress tests, the appearance of the less polar LC-MS compound might correspond to the *cis* racemic species formed in acid aqueous solution. ^1^H NMR of TBZ in acidic aqueous condition would indicate the presence of the *cis* derivative and possibly open intermediate in continuous equilibrium. NMR kinetic studies have also been done over time to monitor *trans*-*cis* interconversion to a fixed pH and/or with temperature variation. A fixed pH kinetic study at room temperature showed a very fast interconversion after addition of acid and no longer evolved significantly over time (see Figure S7 in Supplementary Materials). Variable temperature kinetic study showed total disappearance of signals at 6.51/6.40 ppm and partial disappearance of signals at 6.56/6.41 ppm with general low field shifting (see Figure S8 in Supplementary Materials). Attempts to isolate in a solid state the hypothetical *cis*-TBZ isomer by extraction in organic phase after alkalinization were unsuccessful. Samples obtained from these extractions and checked in LC-MS showed intact *trans*-TBZ and did not show the presence of the more lipophilic impurity. The *cis*-TBZ isomer is probably unstable (kinetic product) and converts to the *trans*-TBZ isomer (thermodynamic product). In our hand, the *cis*-TBZ isomer is only observable in acid solution regardless of temperature. It is noteworthy that the actual drug for patients is unsalified (base form) and there is probably no *cis*-TBZ isomer in the actual drug for patients.

TBZ was formulated as an orodispersible film for paediatric administration by the solvent casting/evaporation method [15,16]. Because TBZ was poorly soluble in aqueous solution excepted in acidic aqueous condition, the use of aqueous citric acid was required to dissolve and mix TBZ and polymer in solution. Indeed, organic solvent (e.g., ethanol) was not recommended for the paediatric population [24], and citric acid offered other advantages, such as palatability enhancement and plasticizer effect on the formulation [18]. However, as shown in this work, acid aqueous condition for TBZ-loaded ODF formulation generates partial *trans*-TBZ interconversion with *cis*-TBZ. The citric acid in ODF would allow partial interconversion in *cis* form, and this is more or less maintained over time depending on the matrix polymer used (presence or not of hydrogen bonds and good miscibility). We hypothesize that apparition of the opaque area after 3 months in F2 and 6 months in F4 (Table 3) is linked to the transformation of *cis*-TBZ into *trans*-TBZ which recrystallized. Previously, Senta-Loys et al. highlighted TBZ recrystallization from ODF composed of povidone (PVP) and hydroxyethylcellulose (HEC) (see samples F2 and F4, respectively, in this present study) [16]. This recrystallization was theoretically explained by the lack of hydrogen bonding with PVP and no vitrification effect of HEC. Indeed, TBZ has been characterized as amorphous solid dispersion and parameters, such as H-bond [25] and no “anti-plasticization” effect [26], play an important role to decrease molecular mobility and maintain system stability. Because of molecular dispersion of TBZ was suggested [15], LC-MS analyses were investigated. Thus, results obtained in this context showed changes occurred before recrystallization step. Indeed, we assumed the first step of the system reorganization was based on interconversion of *cis*-TBZ with *trans*-TBZ implying molecular mobility of the active pharmaceutical ingredient (API) in the polymer matrix. Extensive CPMAS (Solid-state Cross-Polarization Magic Angle Spinning) ^13^C NMR measurements have also been done on tetrabenazine-loaded films with and without citric acid but did not give further information.

## 5. Conclusions

In the present study, standardization of TBZ behavior under acidic condition was investigated to explain preliminary observations showing an additional peak at higher retention time generated after aqueous acid exposure. Extensive experiments employing the use of NMR spectrometry and LC-MS analyses have led us to propose interconversion of *trans*-TBZ with *cis*-TBZ. However, as the solid-state *cis*-TBZ isomer isolation is impossible and under the hypothesis of interconversion between *trans*-TBZ and *cis*-TBZ, further investigations need to be conducted to confirm it, for example, DFT calculations [27].

## Figures and Tables

**Table 1:** Acidic hydrolytic stress conditions applied for tetrabenazine (TBZ) and ratio (%) of observed TBZ/Unknown in LC-MS.

Stress Condition	Solvent	Time	Temp. (°C)	Visual Observation	*t*_r_ TBZ (min)	*t*_r_ Unknown (min)	Ratio (%)
No	ACN	One week	4	limpid colorless	1.847	-	-
No	ACN + H_2_O	70 h	70	light yellow	1.853	2.336	79:21
Acid	HCl 0.1 M	70 h	70	dark yellow-orange	1.837	2.314	77:23
Acid	HCl 1 M	16 h	70	dark orange	1.977	2.562	84:16
Acid	HCl 1 M	70 h	70	dark brown	1.850	2.332	78:22
Acid	Citric acid 1.5 M	70 h	70	light yellow	1.852	2.334	79:21

**Table 2:** Overview of orodispersible films (ODF) composition. HPMC: Hydroxypropylmethylcellulose; PVP: Povidone; PUL: Pullulan; HEC; Hydroxyethylcellulose.

Formulation	Polymer Matrix (%)	TBZ (%)	Citric Acid (%)	Glycerol (%)	Sorbitol (%)
HPMC-ODF (F1)	50.00	15.00	15.00	12.00	8.00
PVP-ODF (F2)	54.35	16.30	16.30	13.05	-
PUL-ODF (F3)	54.35	16.30	16.30	13.05	-
HEC-ODF (F4)	56.80	17.05	17.05	-	9.10

**Table 3:** Overview of visual observations and % of TBZ (*t*_r_ 2.9–3.3 min) and unknown compound (*t*_r_ 3.7–4.5 min) in LC-MS analyses at *T* = 0 and after 3 and 6 months of stability. LC-MS separation conditions are the same as in Reference [14] (see Section 2.3.1).

Sample	Visual Observation	TBZ (%)	Unknown (%)
ODF at *T* = 0, ambient temperature (*n* = 3)			
F1	Homogeneous, transparent, slightly yellow	78.4 ± 0.9	17.4 ± 0.3
F2	Homogeneous, transparent, no colour	83.2 ± 2.3	14.2 ± 2.2
F3	Homogeneous, transparent, no colour	81.2 ± 0.5	17.2 ± 0.3
F4	Homogeneous, transparent, no colour	82.3 ± 0.9	14.6 ± 0.2
ODF stored at 40 °C, 75% HR after 3 months (*n* = 3)			
F1	Homogeneous, transparent, yellow	74.2 ± 0.7	17.2 ± 0.4
F2	Homogeneous, opaque area, slightly yellow	91.2 ± 0.6	4.4 ± 0.5
F3	Homogenenous, transparent, slightly yellow	79.7 ± 0.3	15.7 ± 0.1
F4	Heterogeneous, opaque area, slightly yellow	81.0 ± 0.3	14.2 ± 0.3
ODF stored at 40 °C, 75% HR after 6 months (*n* = 3)			
F1	Homogeneous, transparent, yellow	80.1 ± 0.1	16.3 ± 0.1
F2	Homogeneous, opaque area, slightly yellow	89.5 ± 0.7	6.5 ± 0.6
F3	Homogenenous, transparent, slightly yellow	84.3 ± 0.1	14.1 ± 0.6
F4	Heterogeneous, opaque area, slightly yellow	92.65 ±0.7	6.0 ± 0.7

## Supplementary Materials

The following data are available online at http://www.mdpi.com/1999-4923/11/1/44/s1. Figure S1: LC-MS analysis; Figure S2: TBZ 500 MHz ^1^H NMR spectra; Figure S3: Expanded TBZ 500 MHz ^1^H NMR spectra; Figure S4: Deuterated TBZ 500 MHz ^1^H NMR spectrum; Figure S5: ROESY experiments; Figure S6. LC-MS data for analyses of TBZ contained in ODF; Figure S7. Kinetic experiments on TBZ in D_2_O/acetic acid at 24 °C; Figure S8. Kinetic experiments on TBZ in D_2_O/citric acid at variable temperatures.
