# Supplementary material for: Behaviour of Tetrabenazine in Acid Medium: Reassessment and Impact on Formulation
Source: Pharmaceutics. 2019 Jan 20;11(1):44. doi: 10.3390/pharmaceutics11010044 (PMC6359103; doi:10.3390/pharmaceutics11010044)
Supplement: Supplementary file 1 [file pharmaceutics-11-00044-s001.pdf]

pharmaceutics

MDPI

Supplementary Materials: Behaviour of Tetrabenazine in Acid Medium: Reassessment and Impact on Formulation

Laurent Ettouati, Zoé Senta-Loys, Sandrine Bourgeois, Bernard Fenet, Marc Le Borgne and Hatem Fessi

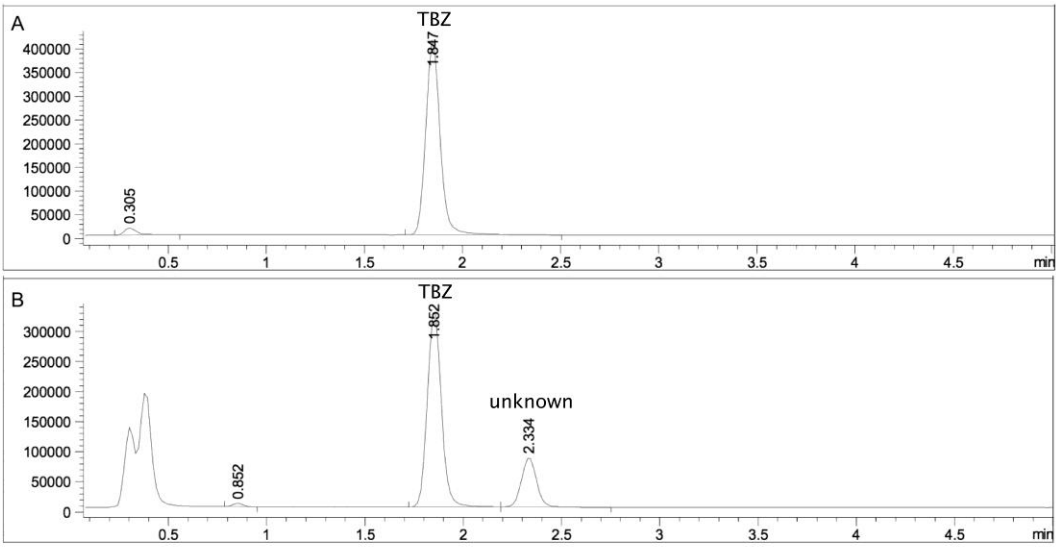

Figure S1. LC-MS analysis. (**A**) SIM chromatogram of TBZ sample. (**B**) Chromatogram of TBZ sample treated with acid citric 1.5 M for 70 h at 70 °C.

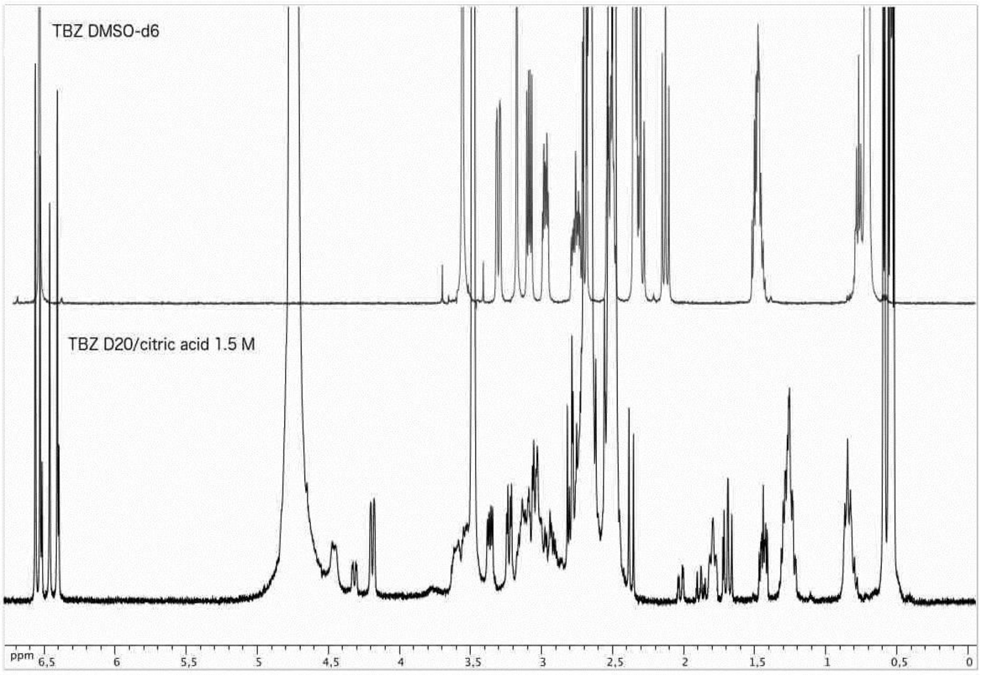

Figure S2. TBZ 500 MHz ^{1}H NMR spectra. Top: TBZ 500 MHz ^{1}H NMR spectrum in DMSO-d_6 solution. Bottom: TBZ 500 MHz ^{1}H NMR spectrum in citric acid 1.5 M / D_2O solution.

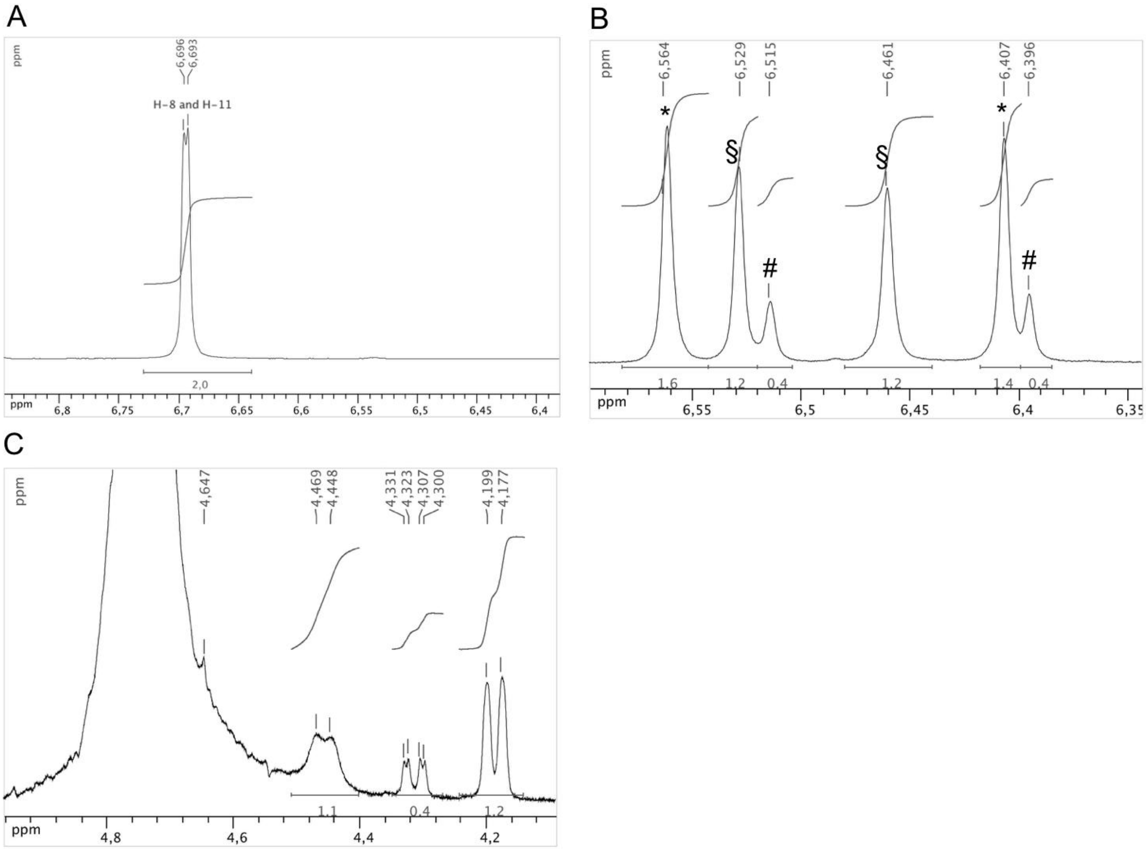

Figure S3. Expanded 500 MHz ^{1}H NMR spectra of TBZ. (**A**) Expanded area between 6.35 and 6.85 ppm in DMSO-d6 solution. (**B**) Expanded area between 6.35 and 6.6 ppm in in citric acid 1.5 M / D_2O solution. (**C**) Expanded area between 4.1 and 5 ppm in citric acid 1.5 M / D_2O solution.

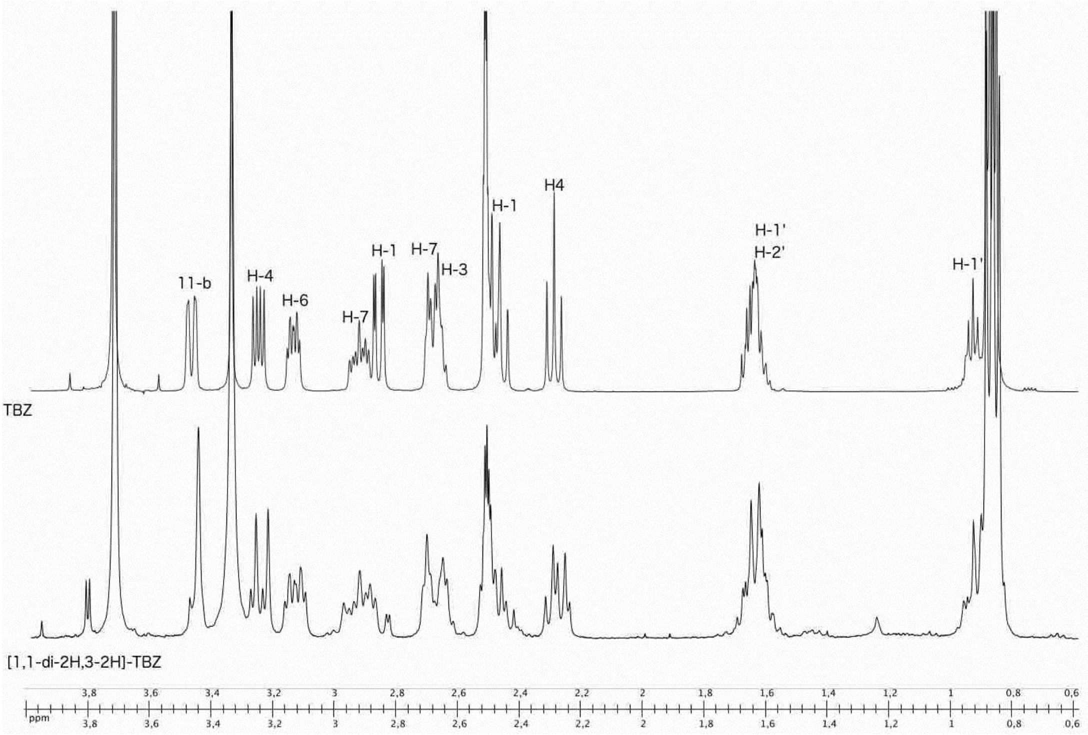

Figure S4. Deuterated TBZ 500 MHz ^{1}H NMR spectrum. Top: TBZ 500 MHz ^{1}H NMR spectrum in DMSO-d6 with protons assignments. Bottom: [1,1-di-^{2}H,3-^{2}H]-TBZ ^{1}H NMR spectrum in DMSO-d6.

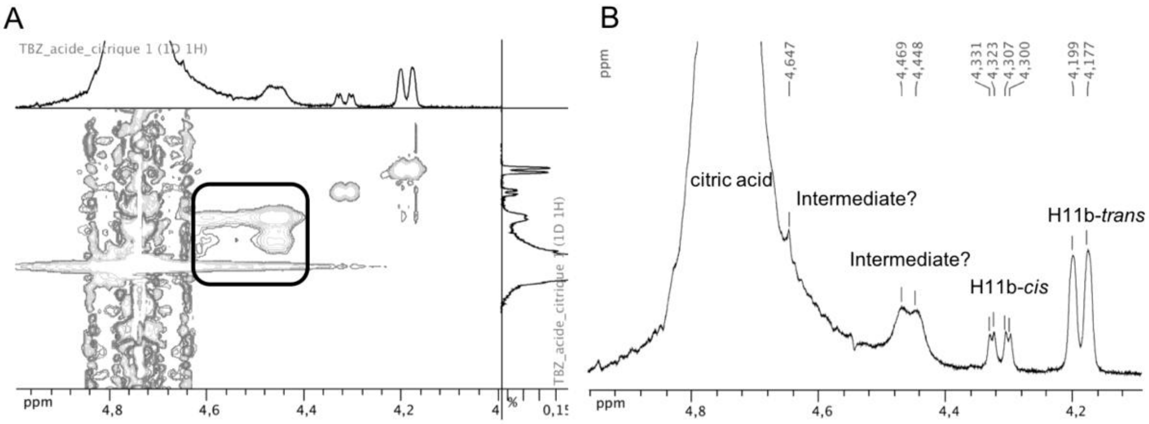

Figure S5. ROESY Experiments. (**A**) ROESY spectrum expansion between 4 and 5 ppm of TBZ in citric acid 1.5 M / D_2O solution. Correlation peaks from the ROE effect are opposite to diagonal peaks and of non-zero intensity. On the other hand, correlation peaks resulting from a chemical exchange phenomenon as indicated by the black box are of the same signs as the diagonal. (**B**) TBZ 500 MHz ^1H NMR spectrum expansion between 4 and 5 ppm in citric acid 1.5 M / D_2O solution with hypothetical assignments.

Reference tetrabenazine LC-MS data

Data File C:\CHEM32\1\DATA\RECHERCHE\TBZ\TBZ 2015-06-18 16-52-22\1DA-0201.D
Sample Name: TBZ REF

===

Acq. Operator : LT Seq. Line : 2
Acq. Instrument : LCMS Location : P1-D-01
Injection Date : 6/18/2015 4:59:49 PM Inj : 1
Inj Volume : 0.100 µl
Acq. Method : C:\CHEM32\1\DATA\RECHERCHE\TBZ\TBZ 2015-06-18 16-52-22\TBZISO_45ACN_55TP4.
Last changed : 5/19/2015 3:39:36 PM by LE-MR
Analysis Method : C:\CHEM32\1\DATA\RECHERCHE\TBZ\TBZ 2015-06-18 16-52-22\1DA-0201.D\DA.M (TBZISO_45ACN_55TP4.5.M, From Data File)
Last changed : 5/19/2015 3:39:36 PM by LE-MR

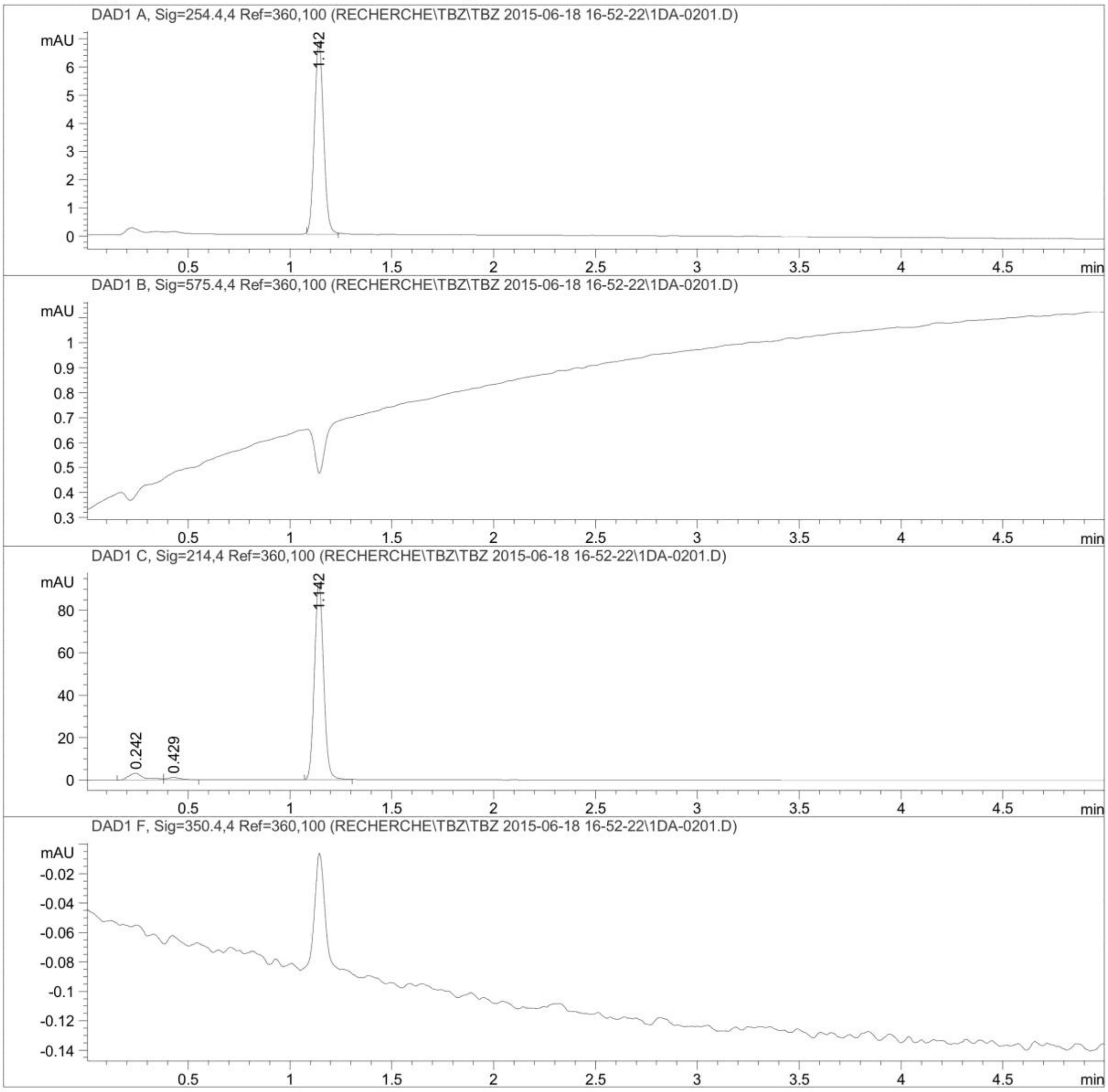

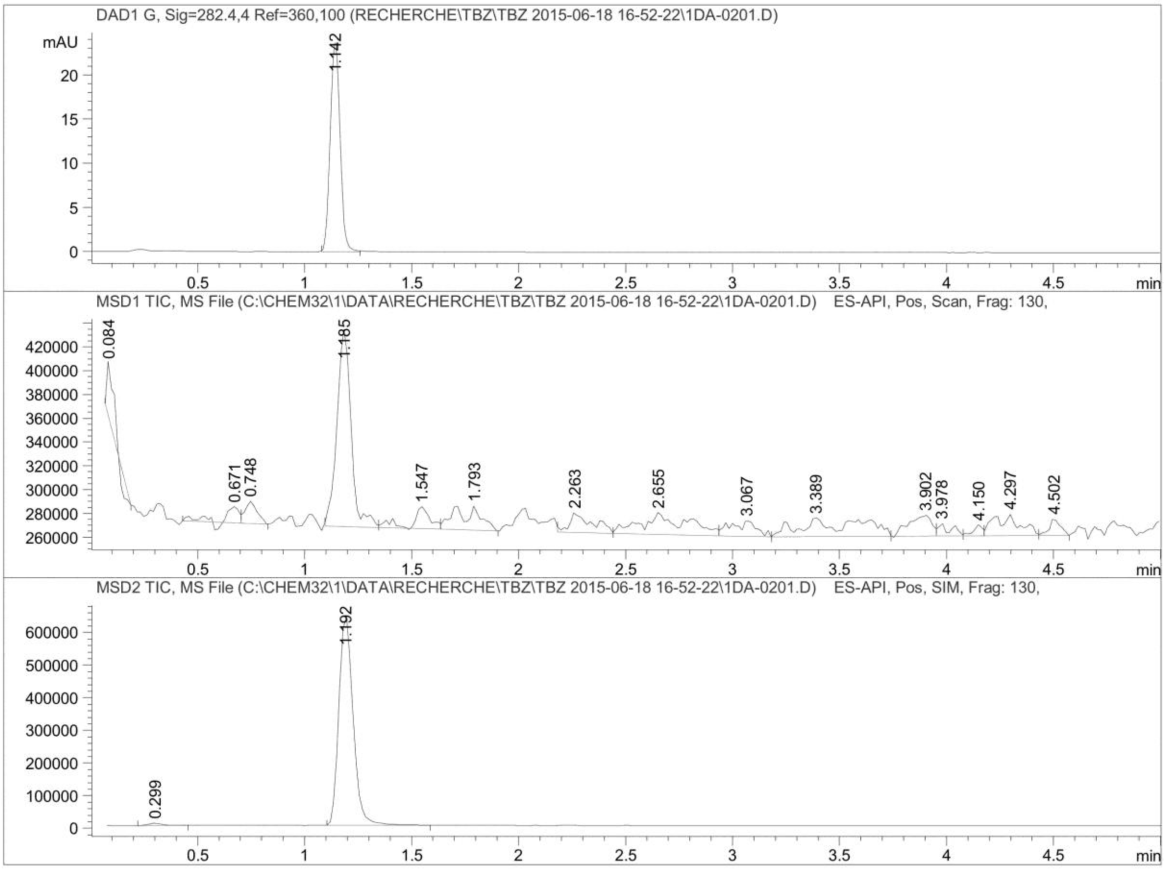

===

Area Percent Report

===

Sorted By : Signal
Multiplier : 1.0000
Dilution : 1.0000
Use Multiplier & Dilution Factor with ISTDs

Signal 1: DAD1 A, Sig=254.4,4 Ref=360,100

Peak #	RetTime [min]	Type	Width [min]	Area [mAU*s]	Height [mAU]	Area %
1	1.142	BB	0.0487	21.73563	6.90487	100.0000

Totals : 21.73563 6.90487

Signal 2: DAD1 B, Sig=575.4,4 Ref=360,100

Signal 3: DAD1 C, Sig=214,4 Ref=360,100

Peak #	RetTime [min]	Type	Width [min]	Area [mAU*s]	Height [mAU]	Area %
1	0.242	BB	0.0757	16.76622	3.17015	5.2347
2	0.429	BB	0.0648	5.53361	1.22315	1.7277
3	1.142	BB	0.0490	297.98877	93.91043	93.0376

Totals : 320.28860 98.30373

Signal 4: DAD1 F, Sig=350.4,4 Ref=360,100

Signal 5: DAD1 G, Sig=282.4,4 Ref=360,100

Peak #	RetTime [min]	Type	Width [min]	Area [mAU*s]	Height [mAU]	Area %
1	1.142	BB	0.0488	75.34365	23.85576	100.0000

Totals : 75.34365 23.85576

Signal 6: MSD1 TIC, MS File

Peak #	RetTime [min]	Type	Width [min]	Area	Height	Area %
1	0.084	BB	0.0273	7.71464e4	4.70234e4	3.1790
2	0.671	BV	0.0690	5.93679e4	1.38193e4	2.4464
3	0.748	VV	0.0589	7.27781e4	1.87602e4	2.9990
4	1.185	BV	0.0672	7.62484e5	1.65897e5	31.4203
5	1.547	VV	0.0837	1.01186e5	1.85209e4	4.1697
6	1.793	VV	0.1319	1.61832e5	2.04526e4	6.6687
7	2.263	BV	0.1191	1.21570e5	1.70120e4	5.0096
8	2.655	VV	0.2378	2.64422e5	1.85321e4	10.8963
9	3.067	VV	0.1345	1.09935e5	1.36251e4	4.5302
10	3.389	VB	0.2285	2.93184e5	1.59176e4	12.0815
11	3.902	BV	0.1057	1.30836e5	1.74420e4	5.3914
12	3.978	VV	0.0653	4.19986e4	1.07182e4	1.7307
13	4.150	VV	0.0469	2.67144e4	9493.25684	1.1008
14	4.297	VV	0.1251	1.52027e5	1.81234e4	6.2647
15	4.502	VV	0.0590	5.12433e4	1.44674e4	2.1116

Totals : 2.42673e6 4.19805e5

Signal 7: MSD2 TIC, MS File

Peak #	RetTime [min]	Type	Width [min]	Area	Height	Area %
1	0.299	BB	0.0709	3.02532e4	6688.86475	1.0077
2	1.192	BB	0.0718	2.97205e6	6.46132e5	98.9923

Totals : 3.00231e6 6.52820e5

===

*** End of Report ***

Stability of F1 sample in LC-MS at 1 month

```
Data File C:\CHEM32\1\DATA\RECHERCHE\TBZ\TBZ 2015-12-14 12-07-27\1BE-1801.D
Sample Name: F1-stab1mois

    =====================================================================
    Acq. Operator   : LE-ZSL                          Seq. Line :  18
    Acq. Instrument : LCMS                             Location : P1-B-05
    Injection Date  : 12/14/2015 2:26:00 PM                 Inj :   1
                                                     Inj Volume : 0.100 µl
    Acq. Method     : C:\CHEM32\1\DATA\RECHERCHE\TBZ\TBZ 2015-12-14 12-07-27\TBZISO_45ACN_55TP4.
    Last changed    : 12/14/2015 11:04:54 AM by LE-ZSL
    Analysis Method : C:\CHEM32\1\DATA\RECHERCHE\TBZ\TBZ 2015-12-14 12-07-27\1BE-1801.D\DA.M (
                      TBZISO_45ACN_55TP4.5.M, From Data File)
    Last changed    : 12/14/2015 11:04:54 AM by LE-ZSL
```

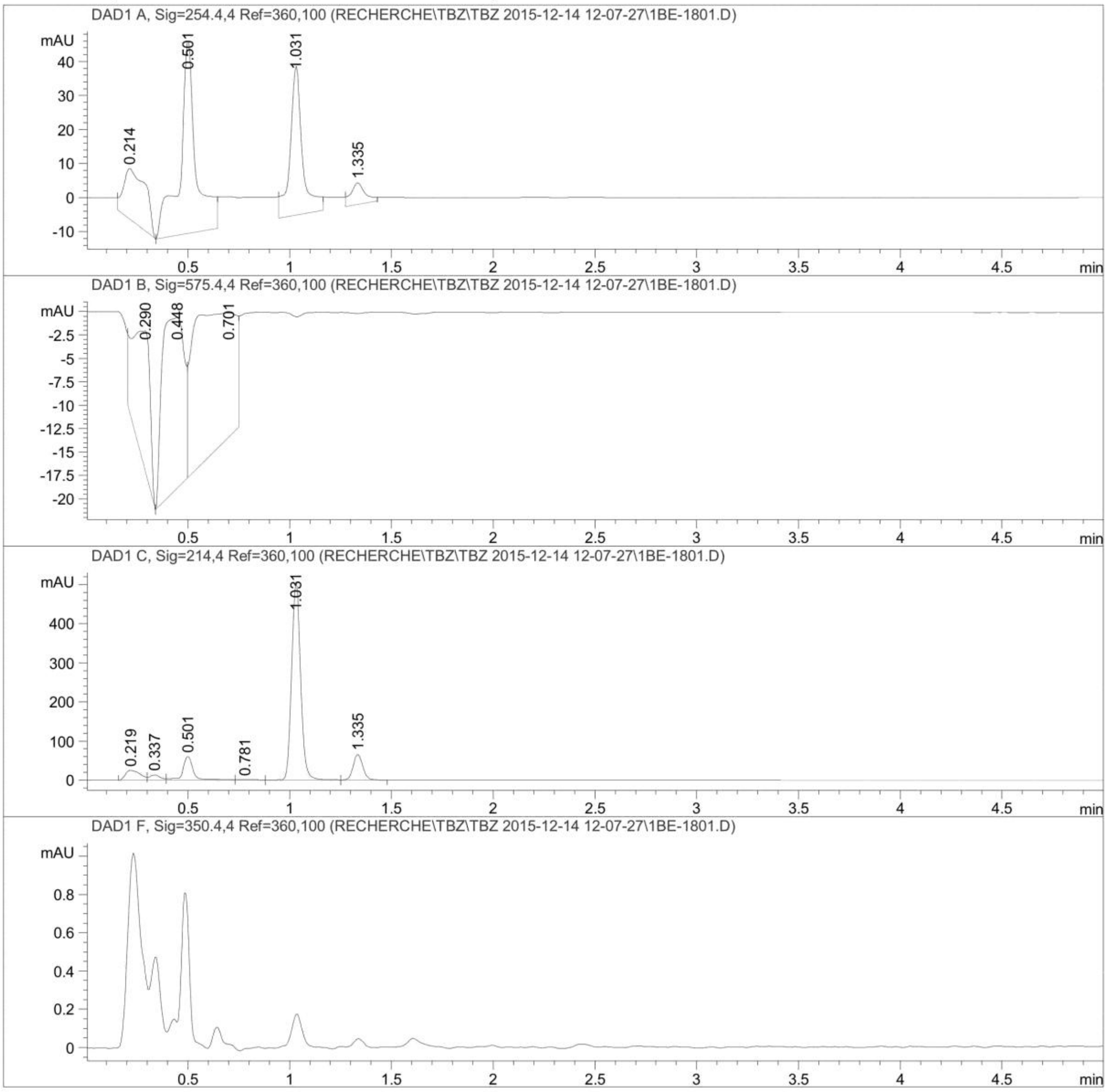

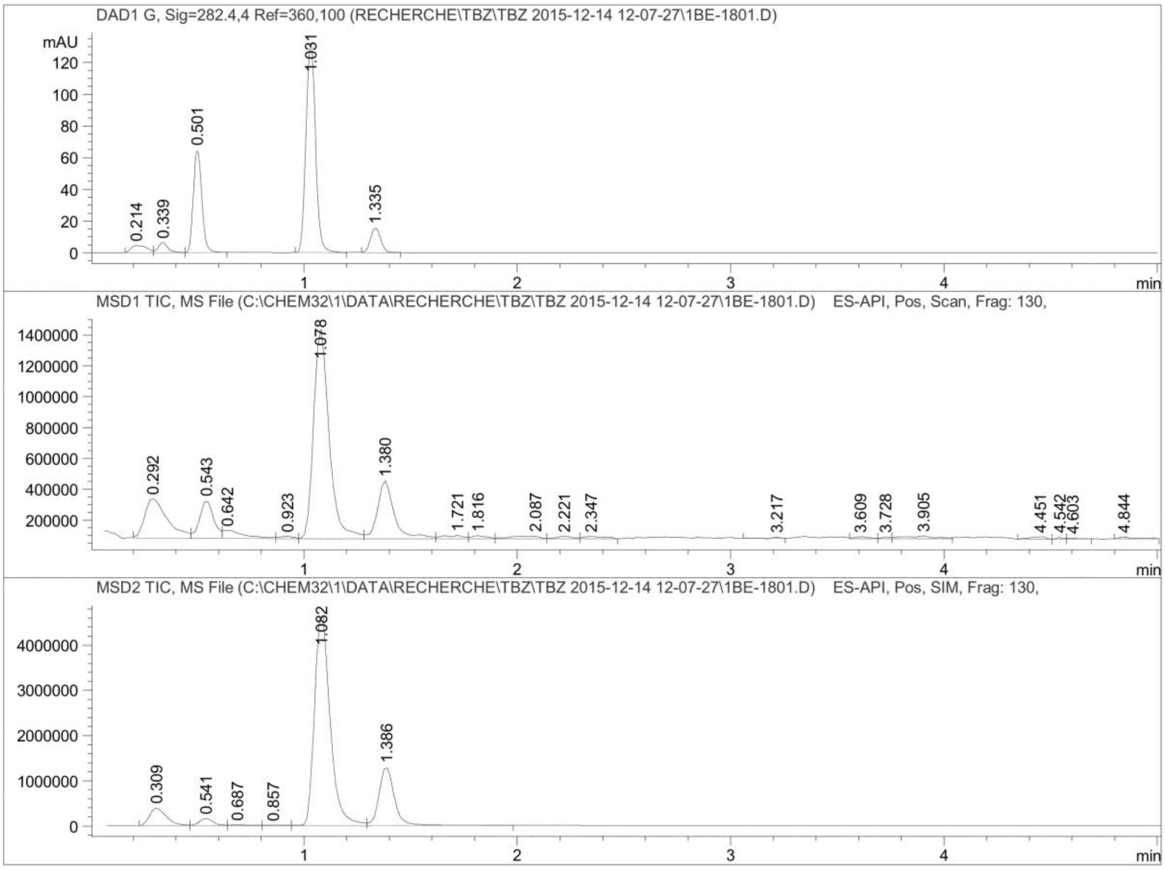

===
Area Percent Report
===

Sorted By : Signal
Multiplier : 1.0000
Dilution : 1.0000
Use Multiplier & Dilution Factor with ISTDs

Signal 1: DAD1 A, Sig=254.4,4 Ref=360,100

Peak #	RetTime [min]	Type	Width [min]	Area [mAU*s]	Height [mAU]	Area %
1	0.214	BV	0.1048	118.35085	14.87868	18.2625
2	0.501	VB	0.0759	311.72409	56.89452	48.1017
3	1.031	BB	0.0616	185.95105	43.77173	28.6938
4	1.335	BB	0.0707	32.02634	6.36608	4.9419

Totals : 648.05233 121.91101

Signal 2: DAD1 B, Sig=575.4,4 Ref=360,100

Peak #	RetTime [min]	Type	Width [min]	Area [mAU*s]	Height [mAU]	Area %
1	0.290	BV	0.0898	84.36113	14.95597	19.1224
2	0.448	VV	0.1239	140.38351	18.21950	31.8211
3	0.701	VV	0.2107	216.41954	13.24105	49.0565

Totals : 441.16418 46.41652

Signal 3: DAD1 C, Sig=214,4 Ref=360,100

Peak #	RetTime [min]	Type	Width [min]	Area [mAU*s]	Height [mAU]	Area %
1	0.219	BV	0.0722	126.82920	25.42974	5.7670
2	0.337	VV	0.0541	47.47918	13.14626	2.1589
3	0.501	VB	0.0532	213.90869	60.49993	9.7266
4	0.781	BV	0.0777	8.28898	1.47221	0.3769
5	1.031	VV	0.0485	1582.61511	506.21436	71.9630
6	1.335	VB	0.0509	220.08665	66.03878	10.0075

Totals : 2199.20782 672.80128

Signal 4: DAD1 F, Sig=350.4,4 Ref=360,100

Signal 5: DAD1 G, Sig=282.4,4 Ref=360,100

Peak #	RetTime [min]	Type	Width [min]	Area [mAU*s]	Height [mAU]	Area %
1	0.214	BV	0.0682	23.15449	4.64357	3.3779
2	0.339	VV	0.0521	20.59518	6.29981	3.0045
3	0.501	VB	0.0448	181.50293	64.61577	26.4785
4	1.031	BB	0.0480	408.87103	132.33722	59.6481
5	1.335	BB	0.0506	51.34834	15.52454	7.4909

Totals : 685.47198 223.42090

Signal 6: MSD1 TIC, MS File

Peak #	RetTime [min]	Type	Width [min]	Area	Height	Area %
1	0.292	BV	0.1088	1.90112e6	2.57610e5	14.1733
2	0.543	VV	0.0710	1.11383e6	2.45679e5	8.3039
3	0.642	VB	0.0990	3.68121e5	5.31254e4	2.7444
4	0.923	BV	0.0648	6.30824e4	1.43784e4	0.4703

Data File C:\CHEM32\1\DATA\RECHERCHE\TBZ\TBZ 2015-12-14 12-07-27\1BE-1801.D
Sample Name: F1-stab1mois

Peak #	RetTime [min]	Type	Width [min]	Area	Height	Area %
5	1.078	VV	0.0771	6.87274e6	1.35891e6	51.2380
6	1.380	VV	0.0814	2.03313e6	3.75124e5	15.1575
7	1.721	VV	0.1095	1.40685e5	2.11206e4	1.0488
8	1.816	VV	0.0652	9.53845e4	1.98319e4	0.7111
9	2.087	VV	0.1176	1.60123e5	1.79601e4	1.1938
10	2.221	VV	0.0809	8.67452e4	1.61294e4	0.6467
11	2.347	VB	0.0994	1.05500e5	1.55046e4	0.7865
12	3.217	BV	0.0389	1.77332e4	8161.44238	0.1322
13	3.609	BV	0.0799	5.96806e4	1.24423e4	0.4449
14	3.728	VV	0.0422	3.06906e4	1.21282e4	0.2288
15	3.905	VV	0.1300	1.89244e5	1.89737e4	1.4109
16	4.451	BV	0.0690	7.49625e4	1.57772e4	0.5589
17	4.542	VV	0.0335	2.27756e4	1.13467e4	0.1698
18	4.603	VB	0.0518	2.12884e4	6847.64600	0.1587
19	4.844	BBA	0.0698	5.65324e4	1.17429e4	0.4215

Totals : 1.34134e7 2.49279e6

Signal 7: MSD2 TIC, MS File

Peak #	RetTime [min]	Type	Width [min]	Area	Height	Area %
1	0.309	BV	0.0821	2.14660e6	3.91548e5	6.4634
2	0.541	VV	0.0699	7.19897e5	1.62254e5	2.1676
3	0.687	VV	0.0917	1.19228e5	1.89146e4	0.3590
4	0.857	VV	0.1001	5.85840e4	7904.08350	0.1764
5	1.082	VV	0.0765	2.36935e7	4.73254e6	71.3412
6	1.386	VB	0.0763	6.47370e6	1.29781e6	19.4924

Totals : 3.32115e7 6.61097e6

==

*** End of Report ***

Stability of F2 sample in LC-MS at 1 month

```
Data File C:\CHEM32\1\DATA\RECHERCHE\TBZ\TBZ 2015-12-14 12-07-27\1BF-1901.D
Sample Name: F2-stab1mois

    =====================================================================
    Acq. Operator    : LE-ZSL                          Seq. Line :  19
    Acq. Instrument  : LCMS                             Location : P1-B-06
    Injection Date   : 12/14/2015 2:32:14 PM                 Inj :   1
                                                      Inj Volume : 0.100 µl
    Acq. Method      : C:\CHEM32\1\DATA\RECHERCHE\TBZ\TBZ 2015-12-14 12-07-27\TBZISO_45ACN_55TP4.
    Last changed     : 12/14/2015 11:04:54 AM by LE-ZSL
    Analysis Method  : C:\CHEM32\1\DATA\RECHERCHE\TBZ\TBZ 2015-12-14 12-07-27\1BF-1901.D\DA.M (
                       TBZISO_45ACN_55TP4.5.M, From Data File)
    Last changed     : 12/14/2015 11:04:54 AM by LE-ZSL
```

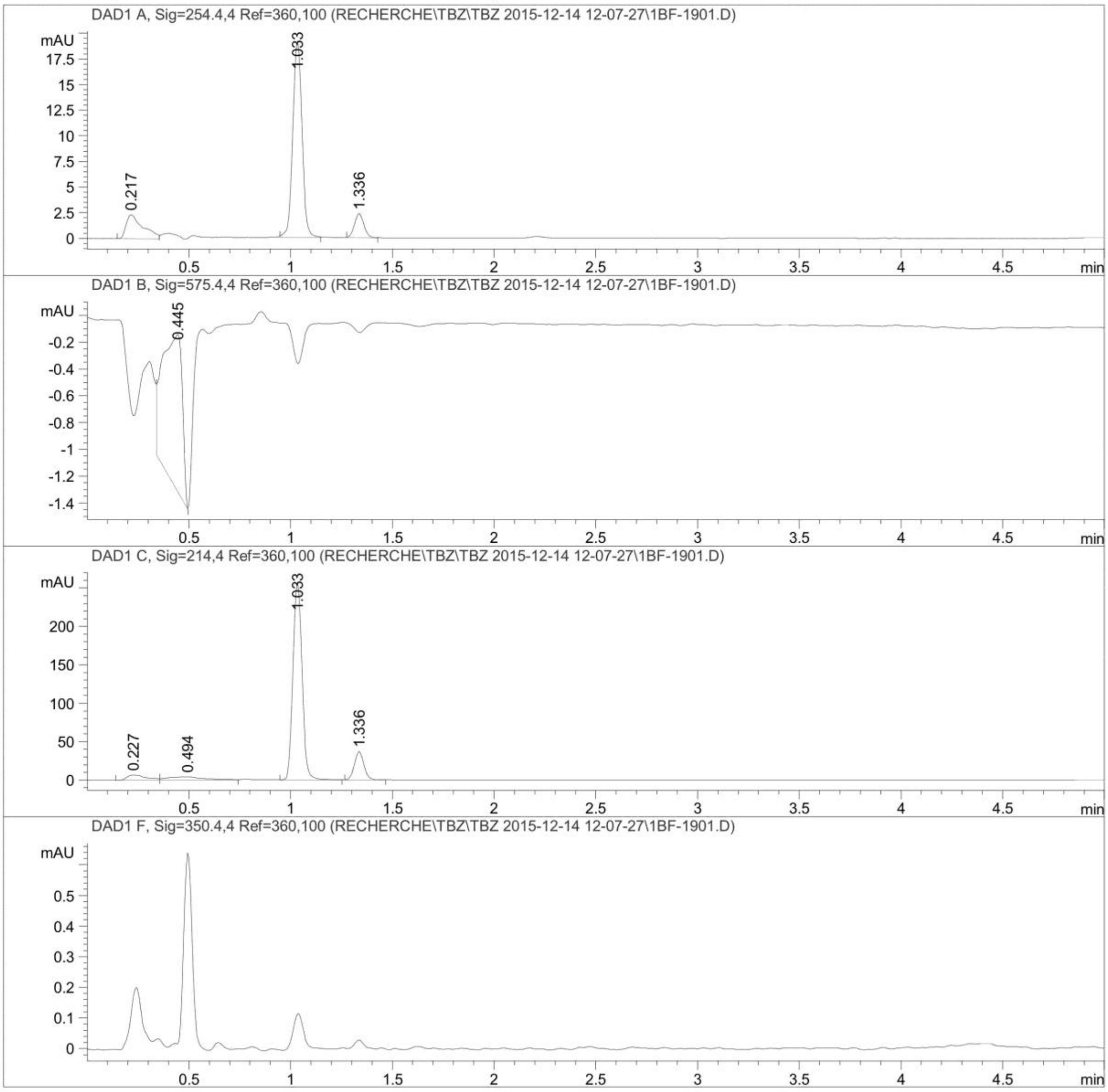

Data File C:\CHEM32\1\DATA\RECHERCHE\TBZ\TBZ 2015-12-14 12-07-27\1BF-1901.D
Sample Name: F2-stab1mois

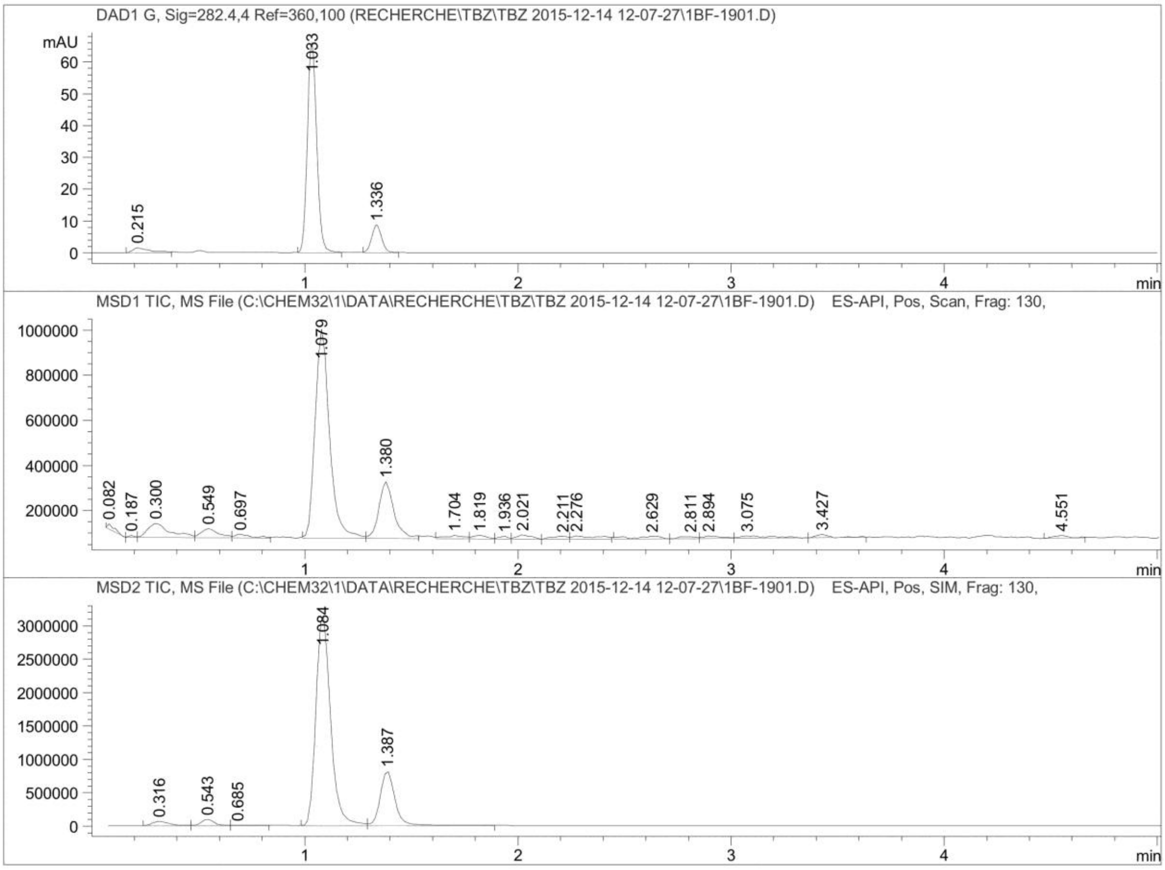

===
Area Percent Report
===

Sorted By : Signal
Multiplier : 1.0000
Dilution : 1.0000
Use Multiplier & Dilution Factor with ISTDs

Signal 1: DAD1 A, Sig=254.4,4 Ref=360,100

Peak #	RetTime [min]	Type	Width [min]	Area [mAU*s]	Height [mAU]	Area %
1	0.217	BV	0.0771	13.14951	2.35484	16.2586
2	1.033	BB	0.0484	59.92933	19.18251	74.0992
3	1.336	BB	0.0501	7.79832	2.38664	9.6422

Totals : 80.87716 23.92398

Signal 2: DAD1 B, Sig=575.4,4 Ref=360,100

Peak #	RetTime [min]	Type	Width [min]	Area [mAU*s]	Height [mAU]	Area %
1	0.445	VV	0.0960	7.67281	1.17852	100.0000

Totals : 7.67281 1.17852

Signal 3: DAD1 C, Sig=214,4 Ref=360,100

Peak #	RetTime [min]	Type	Width [min]	Area [mAU*s]	Height [mAU]	Area %
1	0.227	BV	0.0999	41.29263	6.34894	4.0697
2	0.494	VV	0.1560	52.60461	4.32319	5.1846
3	1.033	BB	0.0501	797.69592	257.54688	78.6188
4	1.336	BB	0.0505	123.04506	37.24385	12.1270

Totals : 1014.63823 305.46286

Signal 4: DAD1 F, Sig=350.4,4 Ref=360,100

Signal 5: DAD1 G, Sig=282.4,4 Ref=360,100

Peak #	RetTime [min]	Type	Width [min]	Area [mAU*s]	Height [mAU]	Area %
1	0.215	BB	0.0806	8.89092	1.51148	3.7071
2	1.033	BB	0.0478	202.27045	65.98585	84.3373
3	1.336	BB	0.0503	28.67367	8.74060	11.9556

Totals : 239.83503 76.23793

Signal 6: MSD1 TIC, MS File

Peak #	RetTime [min]	Type	Width [min]	Area	Height	Area %
1	0.082	BV	0.0374	5.53494e4	2.46520e4	0.7444
2	0.187	VV	0.0272	1.63254e4	1.00051e4	0.2195
3	0.300	VV	0.1122	4.57574e5	6.28068e4	6.1536
4	0.549	VV	0.0815	2.24651e5	4.13423e4	3.0212
5	0.697	VB	0.0707	8.55323e4	1.61891e4	1.1503
6	1.079	BV	0.0735	4.39891e6	9.26524e5	59.1579
7	1.380	VB	0.0756	1.23931e6	2.51391e5	16.6667
8	1.704	BV	0.0991	9.34725e4	1.57195e4	1.2570
9	1.819	VV	0.0785	7.31197e4	1.52868e4	0.9833
10	1.936	VV	0.0471	3.36840e4	1.17186e4	0.4530

Data File C:\CHEM32\1\DATA\RECHERCHE\TBZ\TBZ 2015-12-14 12-07-27\1BF-1901.D
Sample Name: F2-stab1mois

Peak #	RetTime [min]	Type	Width [min]	Area	Height	Area %
11	2.021	VV	0.0771	8.59808e4	1.85867e4	1.1563
12	2.211	VV	0.0723	6.85696e4	1.35818e4	0.9221
13	2.276	VB	0.1151	1.21743e5	1.46918e4	1.6372
14	2.629	BV	0.1394	1.14145e5	1.14055e4	1.5351
15	2.811	VV	0.0807	5.78404e4	1.00573e4	0.7779
16	2.894	VB	0.0743	7.18151e4	1.28464e4	0.9658
17	3.075	BB	0.1254	1.12250e5	1.12616e4	1.5096
18	3.427	BB	0.0717	7.24776e4	1.45577e4	0.9747
19	4.551	BB	0.0667	5.31201e4	1.16800e4	0.7144
Totals :				7.43587e6	1.49430e6	

Signal 7: MSD2 TIC, MS File

Peak #	RetTime [min]	Type	Width [min]	Area	Height	Area %
1	0.316	BV	0.0879	3.56901e5	6.38648e4	1.8045
2	0.543	VV	0.0623	3.90929e5	9.37341e4	1.9765
3	0.685	VB	0.1186	3.54909e4	4985.91992	0.1794
4	1.084	BV	0.0734	1.50897e7	3.18458e6	76.2920
5	1.387	VB	0.0740	3.90583e6	8.15001e5	19.7476
Totals :				1.97788e7	4.16216e6	

==

*** End of Report ***

Stability of F3 sample in LC-MS at 1 month

Data File C:\CHEM32\1\DATA\RECHERCHE\TBZ\TBZ 2015-12-14 12-07-27\1BG-2001.D
Sample Name: F3-stab1mois

===

Acq. Operator : LE-ZSL Seq. Line : 20
Acq. Instrument : LCMS Location : P1-B-07
Injection Date : 12/14/2015 2:38:29 PM Inj : 1
Inj Volume : 0.100 µl
Acq. Method : C:\CHEM32\1\DATA\RECHERCHE\TBZ\TBZ 2015-12-14 12-07-27\TBZISO_45ACN_55TP4.
Last changed : 12/14/2015 11:04:54 AM by LE-ZSL
Analysis Method : C:\CHEM32\1\DATA\RECHERCHE\TBZ\TBZ 2015-12-14 12-07-27\1BG-2001.D\DA.M (TBZISO_45ACN_55TP4.5.M, From Data File)
Last changed : 12/14/2015 11:04:54 AM by LE-ZSL

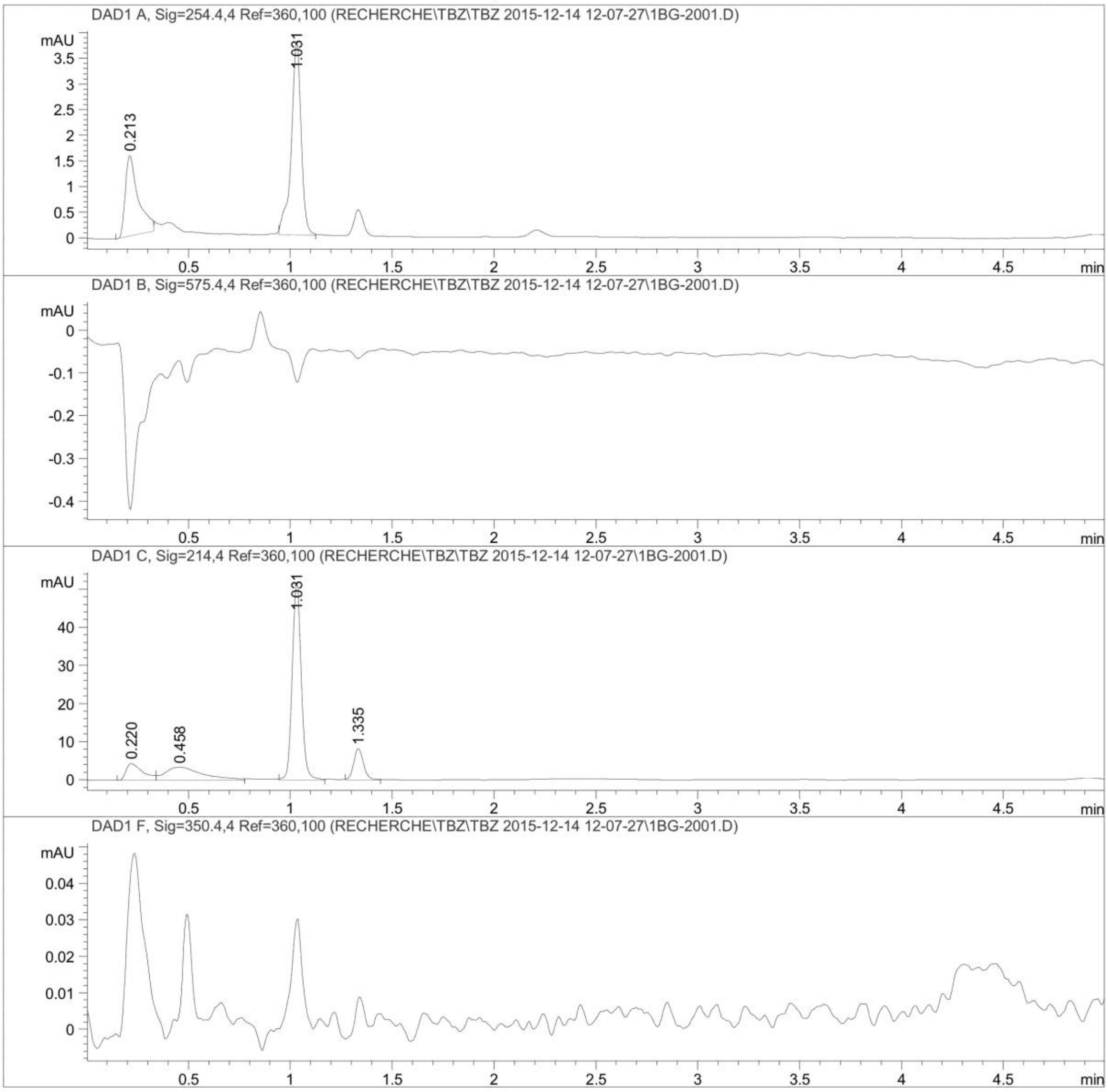

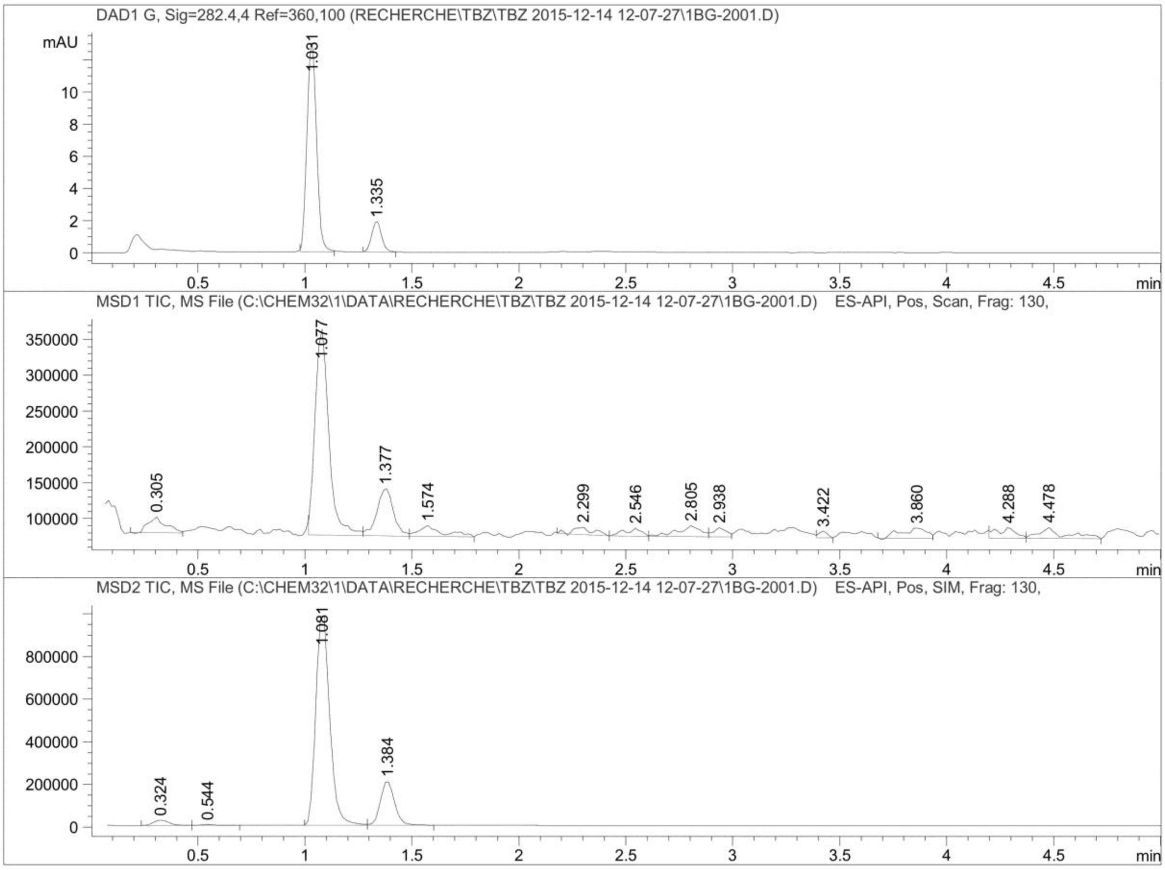

===

Area Percent Report

===

Sorted By : Signal
Multiplier : 1.0000
Dilution : 1.0000
Use Multiplier & Dilution Factor with ISTDs

Signal 1: DAD1 A, Sig=254.4,4 Ref=360,100

Peak #	RetTime [min]	Type	Width [min]	Area [mAU*s]	Height [mAU]	Area %
1	0.213	BB	0.0656	6.95216	1.57175	34.9055
2	1.031	BB	0.0519	12.96492	3.78828	65.0945

Totals : 19.91709 5.36003

Signal 2: DAD1 B, Sig=575.4,4 Ref=360,100

Signal 3: DAD1 C, Sig=214,4 Ref=360,100

Peak #	RetTime [min]	Type	Width [min]	Area [mAU*s]	Height [mAU]	Area %
1	0.220	BV	0.0884	24.80628	4.35916	9.5594
2	0.458	VB	0.1848	43.29214	3.43976	16.6832
3	1.031	BB	0.0506	163.10106	51.97010	62.8532
4	1.335	BB	0.0519	28.29570	8.27604	10.9041
Totals :				259.49517	68.04506	

Signal 4: DAD1 F, Sig=350.4,4 Ref=360,100

Signal 5: DAD1 G, Sig=282.4,4 Ref=360,100

Peak #	RetTime [min]	Type	Width [min]	Area [mAU*s]	Height [mAU]	Area %
1	1.031	BB	0.0499	40.13887	13.04332	86.5595
2	1.335	BB	0.0504	6.23255	1.89557	13.4405
Totals :				46.37142	14.93889	

Signal 6: MSD1 TIC, MS File

Peak #	RetTime [min]	Type	Width [min]	Area	Height	Area %
1	0.305	BV	0.0737	1.26166e5	2.27634e4	4.9478
2	1.077	BV	0.0708	1.30039e6	2.88043e5	50.9965
3	1.377	VV	0.0854	3.56742e5	6.62968e4	13.9901
4	1.574	VV	0.1040	1.16165e5	1.49969e4	4.5555
5	2.299	BV	0.0931	6.87825e4	1.06996e4	2.6974
6	2.546	VV	0.0865	5.82954e4	1.14691e4	2.2861
7	2.805	VV	0.1197	1.10886e5	1.55130e4	4.3485
8	2.938	VV	0.0557	5.31782e4	1.32894e4	2.0854
9	3.422	BV	0.0435	2.55302e4	9778.66699	1.0012
10	3.860	BV	0.1116	1.13856e5	1.42117e4	4.4650
11	4.288	BV	0.1018	9.36363e4	1.53356e4	3.6721
12	4.478	VV	0.1079	1.26334e5	1.56332e4	4.9544
Totals :				2.54996e6	4.98030e5	

Signal 7: MSD2 TIC, MS File

Peak #	RetTime [min]	Type	Width [min]	Area	Height	Area %
1	0.324	BV	0.0787	1.31250e5	2.52782e4	2.3506
2	0.544	VB	0.0687	2.39850e4	5082.92822	0.4296
3	1.081	BV	0.0698	4.47550e6	1.01078e6	80.1529
4	1.384	VB	0.0714	9.52972e5	2.08808e5	17.0670

Totals : 5.58371e6 1.24995e6

*** End of Report ***

Stability of F4 sample in LC-MS at 1 month

Data File C:\CHEM32\1\DATA\RECHERCHE\TBZ\TBZ 2015-12-14 12-07-27\1BH-2101.D
Sample Name: F4-stab1mois

```
=====================================================================
Acq. Operator   : LE-ZSL                          Seq. Line :  21
Acq. Instrument : LCMS                             Location : P1-B-08
Injection Date  : 12/14/2015 2:44:41 PM                 Inj :   1
                                                Inj Volume : 0.100 µl
Acq. Method     : C:\CHEM32\1\DATA\RECHERCHE\TBZ\TBZ 2015-12-14 12-07-27\TBZISO_45ACN_55TP4.
Last changed    : 12/14/2015 11:04:54 AM by LE-ZSL
Analysis Method : C:\CHEM32\1\DATA\RECHERCHE\TBZ\TBZ 2015-12-14 12-07-27\1BH-2101.D\DA.M (
                  TBZISO_45ACN_55TP4.5.M, From Data File)
Last changed    : 12/14/2015 11:04:54 AM by LE-ZSL
```

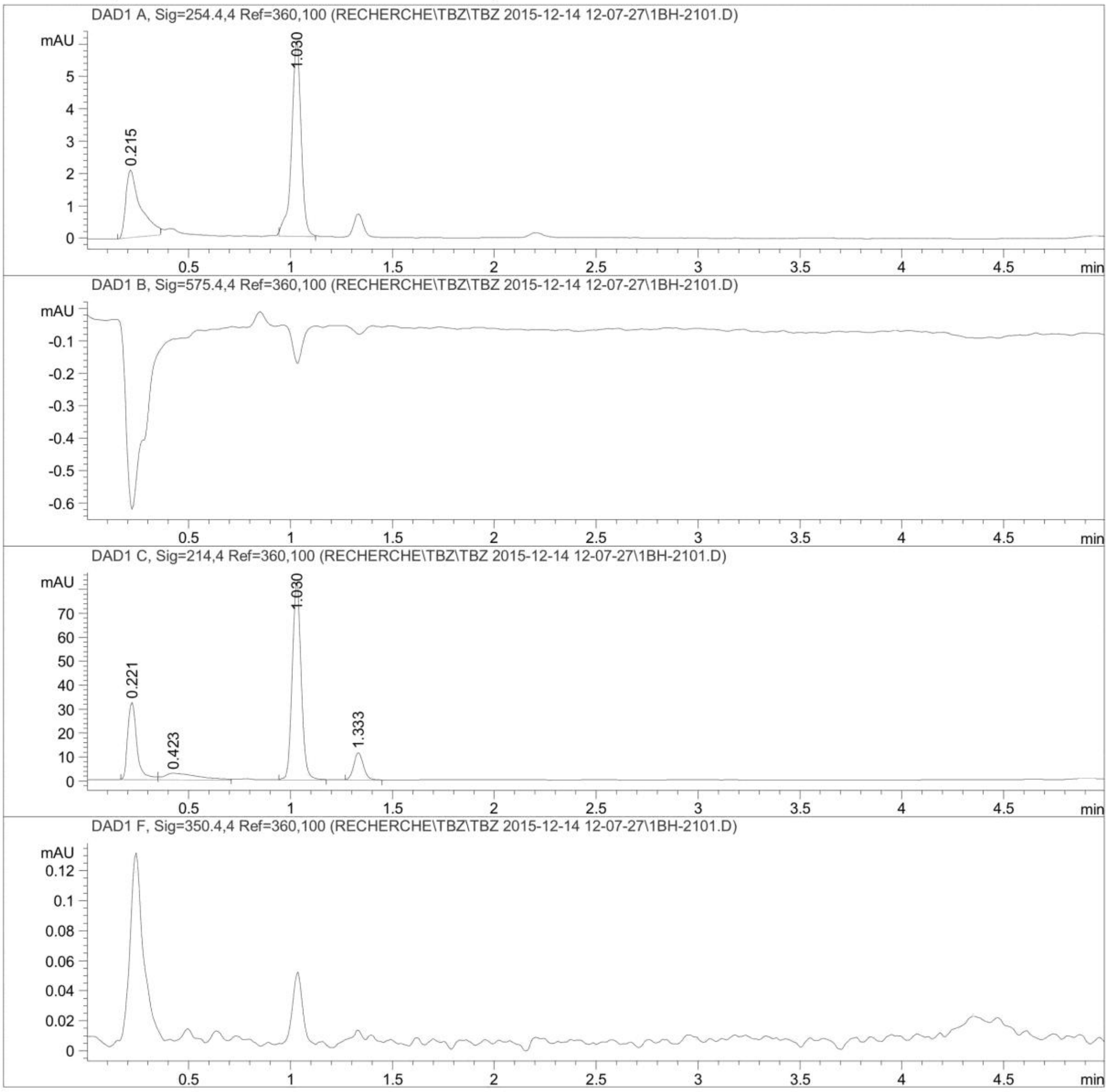

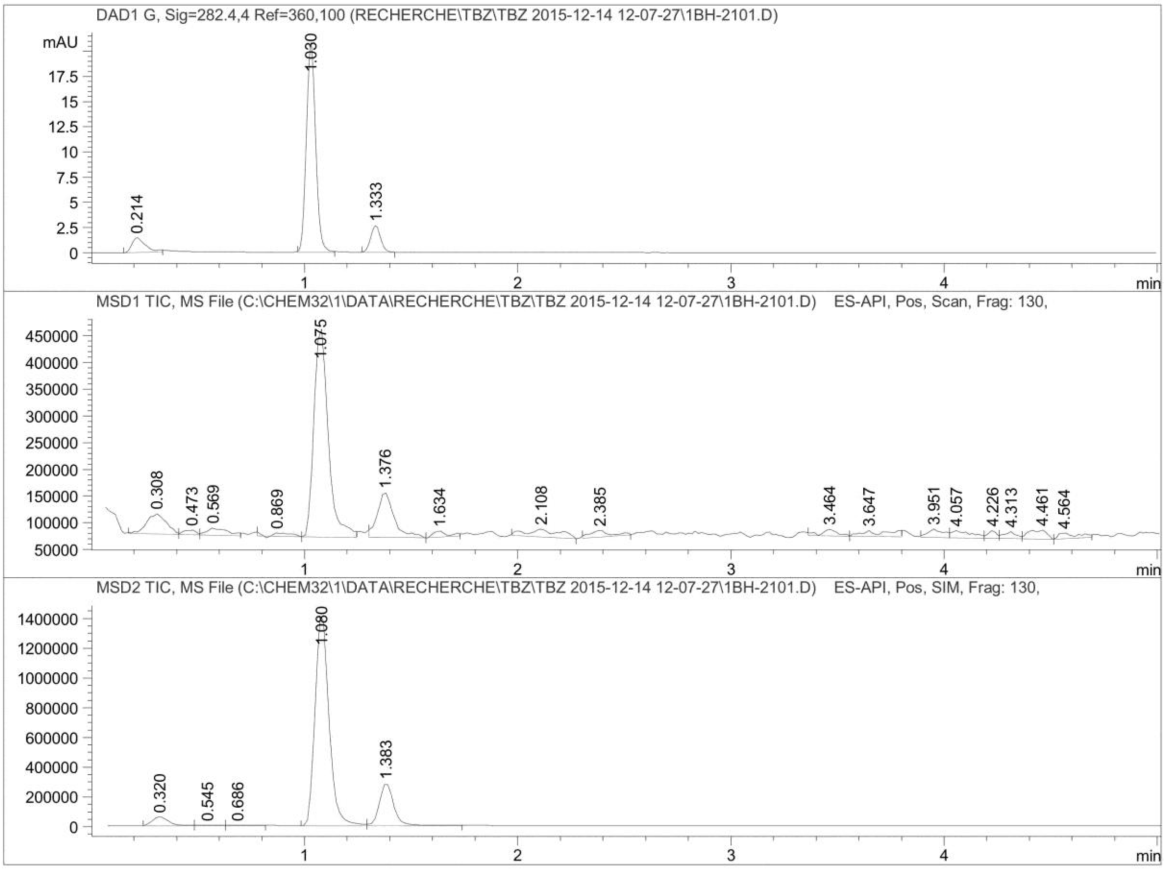

===

Area Percent Report

===

Sorted By : Signal
Multiplier : 1.0000
Dilution : 1.0000
Use Multiplier & Dilution Factor with ISTDs

Signal 1: DAD1 A, Sig=254.4,4 Ref=360,100

Peak #	RetTime [min]	Type	Width [min]	Area [mAU*s]	Height [mAU]	Area %
1	0.215	BB	0.0703	10.48461	2.09783	34.4640
2	1.030	BB	0.0504	19.93726	6.04964	65.5360

Totals : 30.42187 8.14747

Signal 2: DAD1 B, Sig=575.4,4 Ref=360,100

Signal 3: DAD1 C, Sig=214,4 Ref=360,100

Peak #	RetTime [min]	Type	Width [min]	Area [mAU*s]	Height [mAU]	Area %
1	0.221	BV	0.0488	102.01344	32.38387	23.7761
2	0.423	VB	0.1466	32.32064	2.84300	7.5329
3	1.030	BB	0.0502	256.54956	82.70821	59.7935
4	1.333	BB	0.0509	38.17544	11.44939	8.8975
Totals :				429.05909	129.38446	

Signal 4: DAD1 F, Sig=350.4,4 Ref=360,100

Signal 5: DAD1 G, Sig=282.4,4 Ref=360,100

Peak #	RetTime [min]	Type	Width [min]	Area [mAU*s]	Height [mAU]	Area %
1	0.214	BB	0.0629	6.31571	1.44670	8.0034
2	1.030	BB	0.0498	63.92834	20.83736	81.0117
3	1.333	BB	0.0502	8.66841	2.65017	10.9848
Totals :				78.91246	24.93422	

Signal 6: MSD1 TIC, MS File

Peak #	RetTime [min]	Type	Width [min]	Area	Height	Area %
1	0.308	BV	0.0976	2.43254e5	3.78884e4	6.8292
2	0.473	VV	0.0696	3.56555e4	9064.49805	1.0010
3	0.569	VB	0.0949	8.82615e4	1.34211e4	2.4779
4	0.869	BV	0.1027	4.06959e4	6601.48975	1.1425
5	1.075	VB	0.0724	1.82193e6	3.92009e5	51.1492
6	1.376	BV	0.0771	4.60660e5	8.45890e4	12.9327
7	1.634	VB	0.0621	4.85340e4	1.16705e4	1.3626
8	2.108	BB	0.1417	1.53596e5	1.46589e4	4.3121
9	2.385	BB	0.0889	8.05885e4	1.24784e4	2.2625
10	3.464	BV	0.0748	6.49957e4	1.23964e4	1.8247
11	3.647	VB	0.1160	1.01163e5	1.10405e4	2.8401
12	3.951	BV	0.0687	7.98584e4	1.56158e4	2.2420
13	4.057	VV	0.1062	8.54442e4	1.34132e4	2.3988
14	4.226	VV	0.0460	4.06104e4	1.47070e4	1.1401
15	4.313	VV	0.0663	5.14440e4	1.29372e4	1.4442
16	4.461	VV	0.0811	9.94045e4	1.71882e4	2.7907
17	4.564	VB	0.0844	6.58931e4	1.08618e4	1.8499

Totals : 3.56199e6 6.90541e5

Signal 7: MSD2 TIC, MS File

Peak #	RetTime [min]	Type	Width [min]	Area	Height	Area %
1	0.320	BV	0.0849	3.06515e5	5.75014e4	3.7912
2	0.545	VV	0.0813	1.62996e4	2809.67896	0.2016
3	0.686	VB	0.1007	1.23629e4	1655.27429	0.1529
4	1.080	BV	0.0704	6.43858e6	1.43828e6	79.6368
5	1.383	VB	0.0720	1.31118e6	2.83948e5	16.2175

Totals : 8.08494e6 1.78419e6

===

*** End of Report ***

Stability of F1 sample in LC-MS at 3 months

```
Data File C:\CHEM32\1\DATA\RECHERCHE\TBZ\TBZ 2015-12-14 12-07-27\1AB-0201.D
Sample Name: F1-stab3mois-1

    =====================================================================
    Acq. Operator   : LE-ZSL                           Seq. Line :   2
    Acq. Instrument : LCMS                              Location : P1-A-02
    Injection Date  : 12/14/2015 12:21:07 PM                 Inj :   1
                                                      Inj Volume : 0.100 µl
    Acq. Method     : C:\CHEM32\1\DATA\RECHERCHE\TBZ\TBZ 2015-12-14 12-07-27\TBZISO_45ACN_55TP4.
    Last changed    : 12/14/2015 11:04:54 AM by LE-ZSL
    Analysis Method : C:\CHEM32\1\DATA\RECHERCHE\TBZ\TBZ 2015-12-14 12-07-27\1AB-0201.D\DA.M (
                      TBZISO_45ACN_55TP4.5.M, From Data File)
    Last changed    : 12/14/2015 11:04:54 AM by LE-ZSL
```

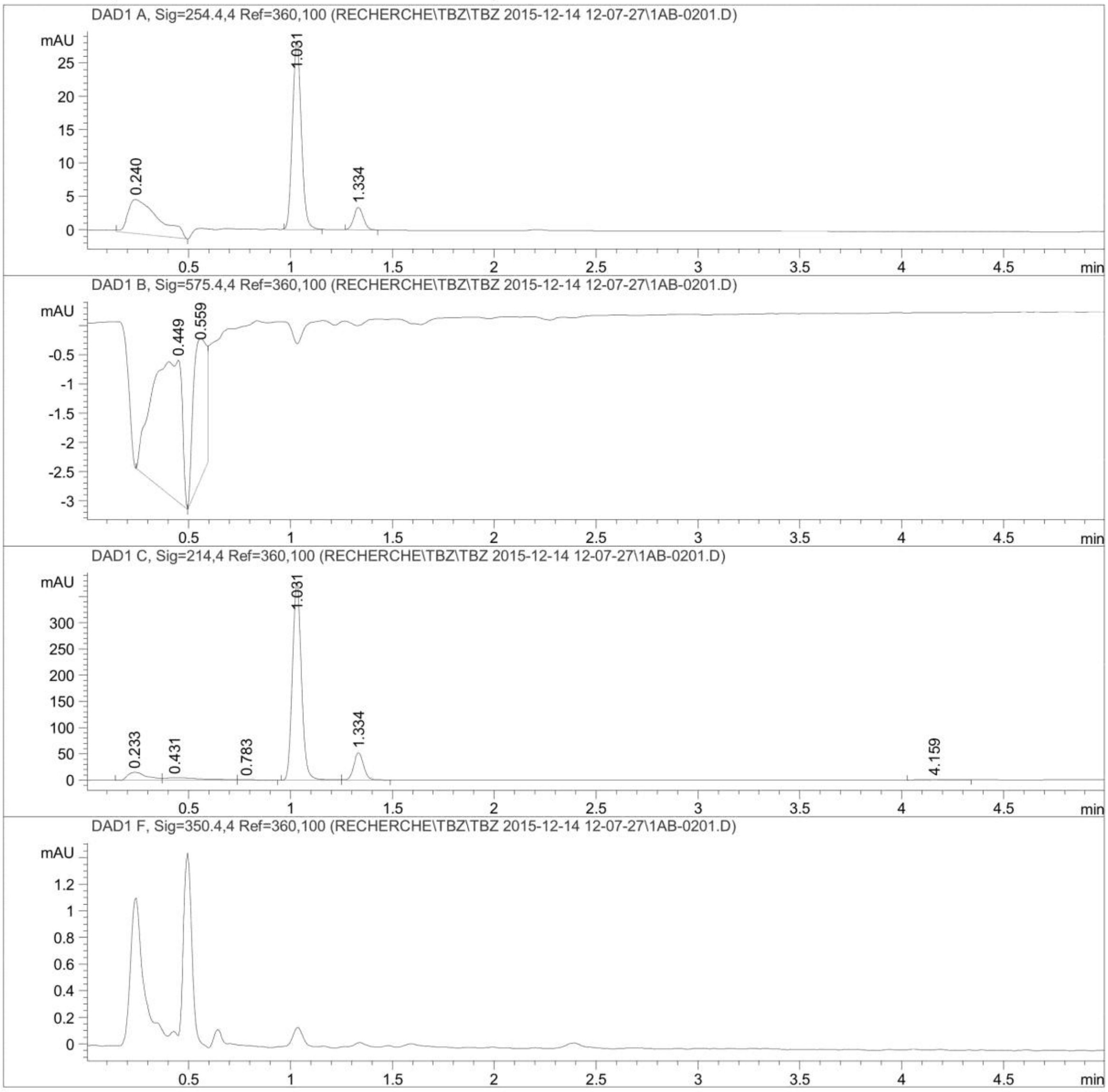

Data File C:\CHEM32\1\DATA\RECHERCHE\TBZ\TBZ 2015-12-14 12-07-27\1AB-0201.D
Sample Name: F1-stab3mois-1

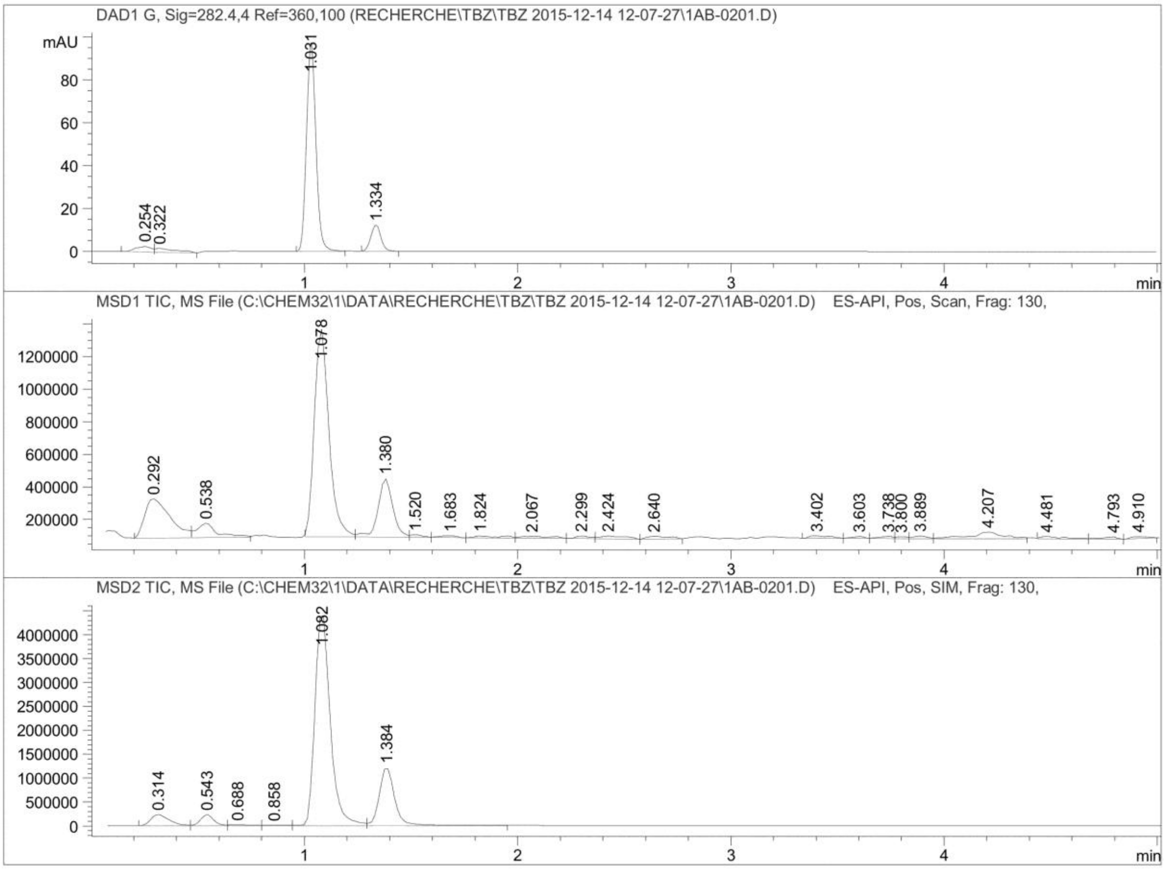

===
Area Percent Report
===

Sorted By : Signal
Multiplier : 1.0000
Dilution : 1.0000
Use Multiplier & Dilution Factor with ISTDs

Signal 1: DAD1 A, Sig=254.4,4 Ref=360,100

Peak #	RetTime [min]	Type	Width [min]	Area [mAU*s]	Height [mAU]	Area %
1	0.240	BV	0.1391	53.98235	5.11810	35.3758
2	1.031	BB	0.0479	87.37914	28.39297	57.2615
3	1.334	BB	0.0526	11.23528	3.39231	7.3627

Totals : 152.59677 36.90338

Signal 2: DAD1 B, Sig=575.4,4 Ref=360,100

Peak #	RetTime [min]	Type	Width [min]	Area [mAU*s]	Height [mAU]	Area %
1	0.449	BV	0.1370	23.63238	2.44092	68.1000
2	0.559	VV	0.0734	11.07010	2.41811	31.9000

Totals : 34.70247 4.85902

Signal 3: DAD1 C, Sig=214,4 Ref=360,100

Peak #	RetTime [min]	Type	Width [min]	Area [mAU*s]	Height [mAU]	Area %
1	0.233	BV	0.1009	98.47347	14.93096	6.4513
2	0.431	VV	0.1731	59.75927	4.73986	3.9150
3	0.783	VB	0.0651	5.51007	1.20973	0.3610
4	1.031	BV	0.0484	1180.28540	378.78094	77.3241
5	1.334	VB	0.0529	174.93803	52.47741	11.4607
6	4.159	BB	0.1099	7.44784	1.03588	0.4879

Totals : 1526.41409 453.17478

Signal 4: DAD1 F, Sig=350.4,4 Ref=360,100

Signal 5: DAD1 G, Sig=282.4,4 Ref=360,100

Peak #	RetTime [min]	Type	Width [min]	Area [mAU*s]	Height [mAU]	Area %
1	0.254	BV	0.0710	13.01834	2.49106	3.5423
2	0.322	VV	0.0915	12.02399	1.76484	3.2718
3	1.031	BB	0.0481	301.90860	97.59741	82.1503
4	1.334	BB	0.0526	40.55666	12.26424	11.0356

Totals : 367.50759 114.11756

Signal 6: MSD1 TIC, MS File

Peak #	RetTime [min]	Type	Width [min]	Area	Height	Area %
1	0.292	BV	0.1187	1.99546e6	2.42557e5	16.4509
2	0.538	VB	0.0953	5.76797e5	8.71985e4	4.7552
3	1.078	BV	0.0738	6.08279e6	1.27491e6	50.1475
4	1.380	VV	0.0745	1.73495e6	3.58855e5	14.3032
5	1.520	VV	0.0601	6.57633e4	1.64842e4	0.5422
6	1.683	VV	0.0958	6.91465e4	1.17687e4	0.5701

Peak #	RetTime [min]	Type	Width [min]	Area	Height	Area %
7	1.824	VV	0.1150	1.01875e5	1.17253e4	0.8399
8	2.067	VB	0.1278	1.24293e5	1.32191e4	1.0247
9	2.299	BV	0.0739	7.65602e4	1.65691e4	0.6312
10	2.424	VV	0.1244	1.43238e5	1.91849e4	1.1809
11	2.640	VV	0.1048	1.14815e5	1.66485e4	0.9466
12	3.402	BB	0.0940	1.05004e5	1.52442e4	0.8657
13	3.603	BV	0.0561	5.11891e4	1.40405e4	0.4220
14	3.738	VV	0.0693	5.78816e4	1.32078e4	0.4772
15	3.800	VV	0.0512	3.81898e4	1.24372e4	0.3148
16	3.889	VV	0.0704	7.12211e4	1.59046e4	0.5872
17	4.207	VB	0.1517	4.59873e5	4.15828e4	3.7913
18	4.481	BV	0.0981	1.18323e5	1.63465e4	0.9755
19	4.793	VV	0.0863	7.16016e4	1.38360e4	0.5903
20	4.910	VBA	0.0779	7.08287e4	1.28425e4	0.5839
Totals :				1.21298e7	2.22456e6	

Signal 7: MSD2 TIC, MS File

Peak #	RetTime [min]	Type	Width [min]	Area	Height	Area %
1	0.314	BV	0.0919	1.37556e6	2.31904e5	4.4890
2	0.543	VV	0.0670	9.61623e5	2.29786e5	3.1381
3	0.688	VV	0.0848	1.25768e5	2.20246e4	0.4104
4	0.858	VV	0.1030	5.23715e4	6833.12744	0.1709
5	1.082	VV	0.0756	2.21134e7	4.49211e6	72.1643
6	1.384	VB	0.0756	6.01442e6	1.22051e6	19.6273
Totals :				3.06431e7	6.20317e6	

===

*** End of Report ***

Stability of F2 sample in LC-MS at 3 months

```
Data File C:\CHEM32\1\DATA\RECHERCHE\TBZ\TBZ 2015-12-14 12-07-27\1AE-0601.D
Sample Name: F2-stab3mois-1

    =====================================================================
    Acq. Operator    : LE-ZSL                          Seq. Line :   6
    Acq. Instrument  : LCMS                             Location : P1-A-05
    Injection Date   : 12/14/2015 12:52:17 PM                Inj :   1
                                                      Inj Volume : 0.100 µl
    Acq. Method      : C:\CHEM32\1\DATA\RECHERCHE\TBZ\TBZ 2015-12-14 12-07-27\TBZISO_45ACN_55TP4.
    Last changed     : 12/14/2015 11:04:54 AM by LE-ZSL
    Analysis Method  : C:\CHEM32\1\DATA\RECHERCHE\TBZ\TBZ 2015-12-14 12-07-27\1AE-0601.D\DA.M (
                       TBZISO_45ACN_55TP4.5.M, From Data File)
    Last changed     : 12/14/2015 11:04:54 AM by LE-ZSL
```

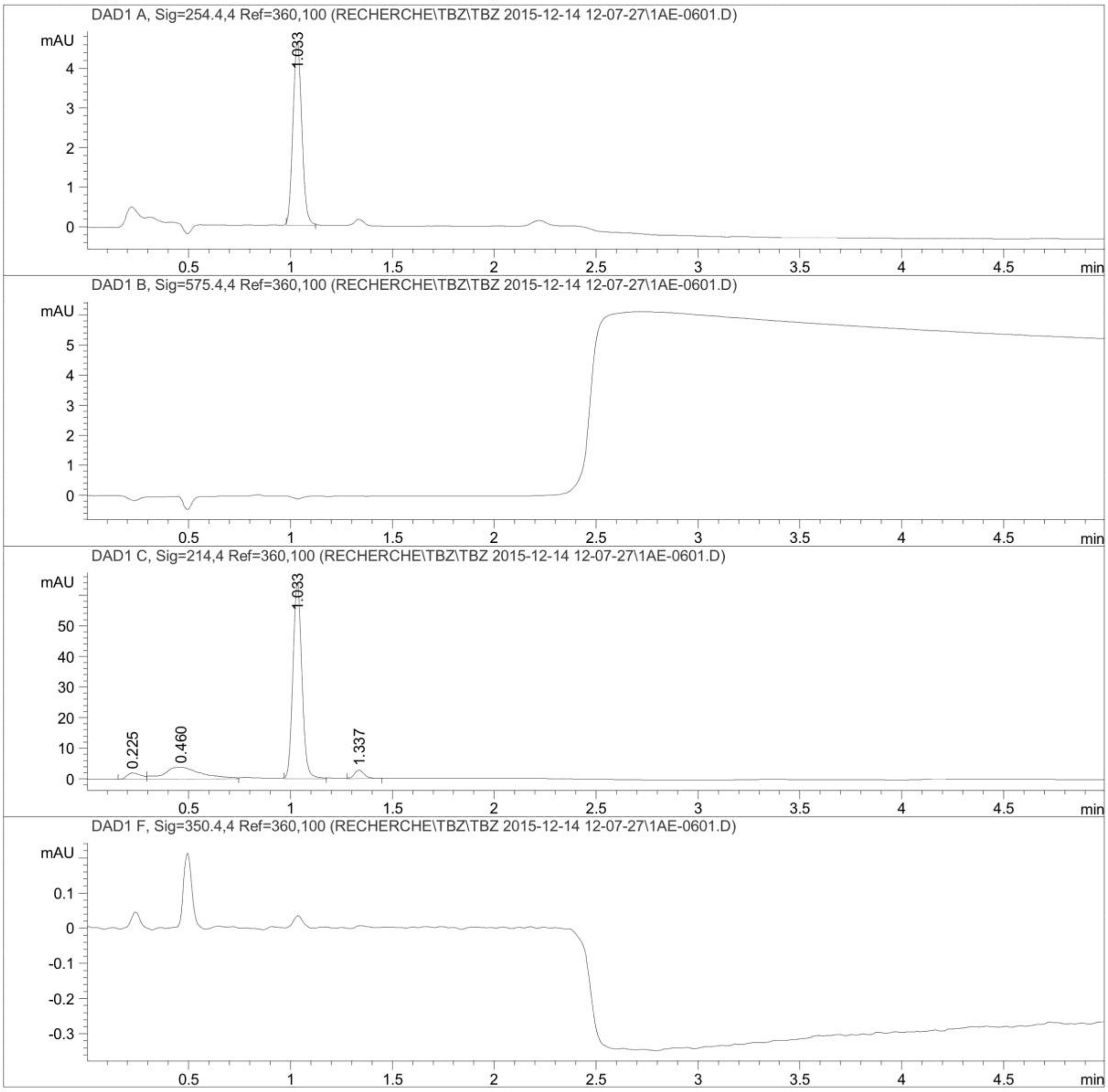

Data File C:\CHEM32\1\DATA\RECHERCHE\TBZ\TBZ 2015-12-14 12-07-27\1AE-0601.D
Sample Name: F2-stab3mois-1

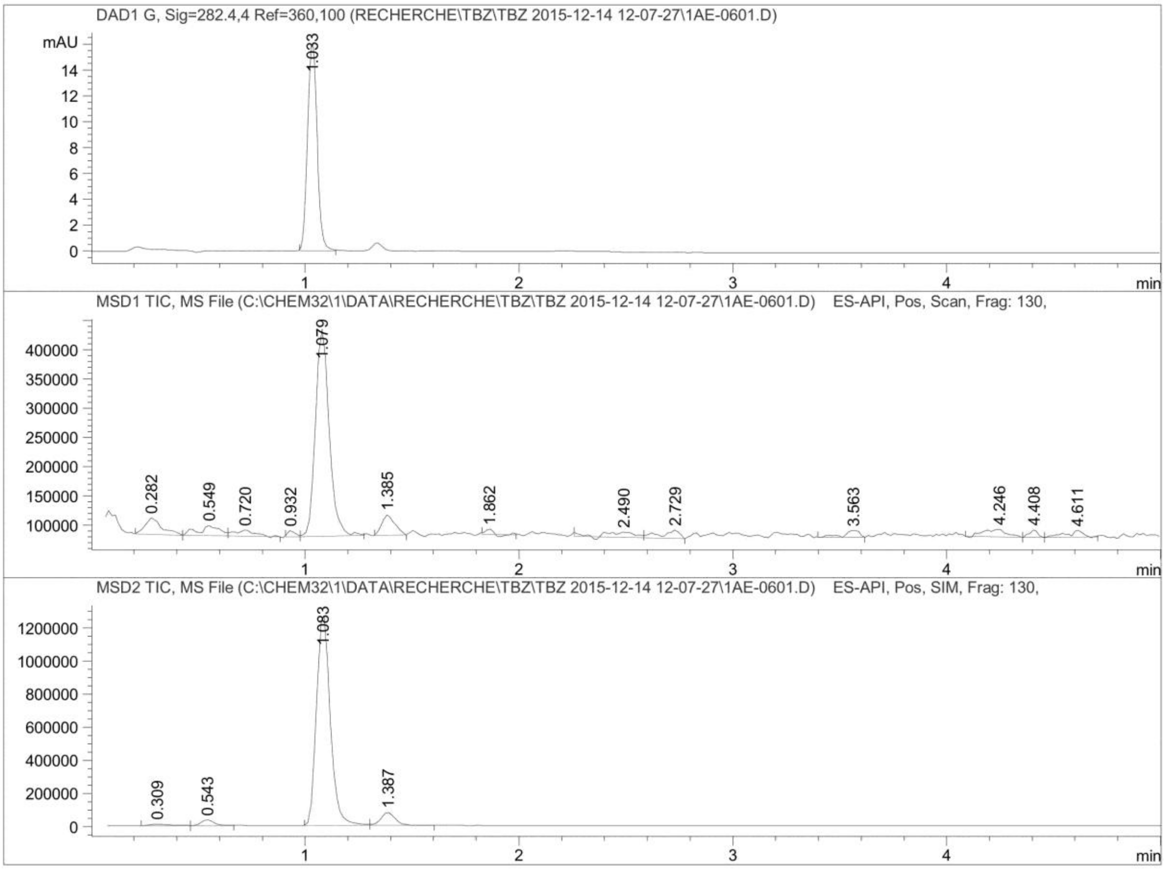

===

Area Percent Report

===

Sorted By : Signal
Multiplier : 1.0000
Dilution : 1.0000
Use Multiplier & Dilution Factor with ISTDs

Signal 1: DAD1 A, Sig=254.4,4 Ref=360,100

Peak #	RetTime [min]	Type	Width [min]	Area [mAU*s]	Height [mAU]	Area %
1	1.033	BB	0.0477	14.31103	4.68170	100.0000

Totals : 14.31103 4.68170

Data File C:\CHEM32\1\DATA\RECHERCHE\TBZ\TBZ 2015-12-14 12-07-27\1AE-0601.D
Sample Name: F2-stab3mois-1

Signal 7: MSD2 TIC, MS File

Peak #	RetTime [min]	Type	Width [min]	Area	Height	Area %
1	0.309	BV	0.0785	5.34606e4	9606.53320	0.8478
2	0.543	VB	0.0604	1.37750e5	3.43607e4	2.1845
3	1.083	BV	0.0702	5.73514e6	1.28624e6	90.9511
4	1.387	VB	0.0737	3.79390e5	7.96198e4	6.0166
Totals :				6.30574e6	1.40983e6	

==

*** End of Report ***

Stability of F3 sample in LC-MS at 3 months

Data File C:\CHEM32\1\DATA\RECHERCHE\TBZ\TBZ 2015-12-14 12-07-27\1AH-1001.D
Sample Name: F3-stab3mois-1

===

Acq. Operator : LE-ZSL Seq. Line : 10
Acq. Instrument : LCMS Location : P1-A-08
Injection Date : 12/14/2015 1:23:34 PM Inj : 1
Inj Volume : 0.100 µl
Acq. Method : C:\CHEM32\1\DATA\RECHERCHE\TBZ\TBZ 2015-12-14 12-07-27\TBZISO_45ACN_55TP4.
Last changed : 12/14/2015 11:04:54 AM by LE-ZSL
Analysis Method : C:\CHEM32\1\DATA\RECHERCHE\TBZ\TBZ 2015-12-14 12-07-27\1AH-1001.D\DA.M (TBZISO_45ACN_55TP4.5.M, From Data File)
Last changed : 12/14/2015 11:04:54 AM by LE-ZSL

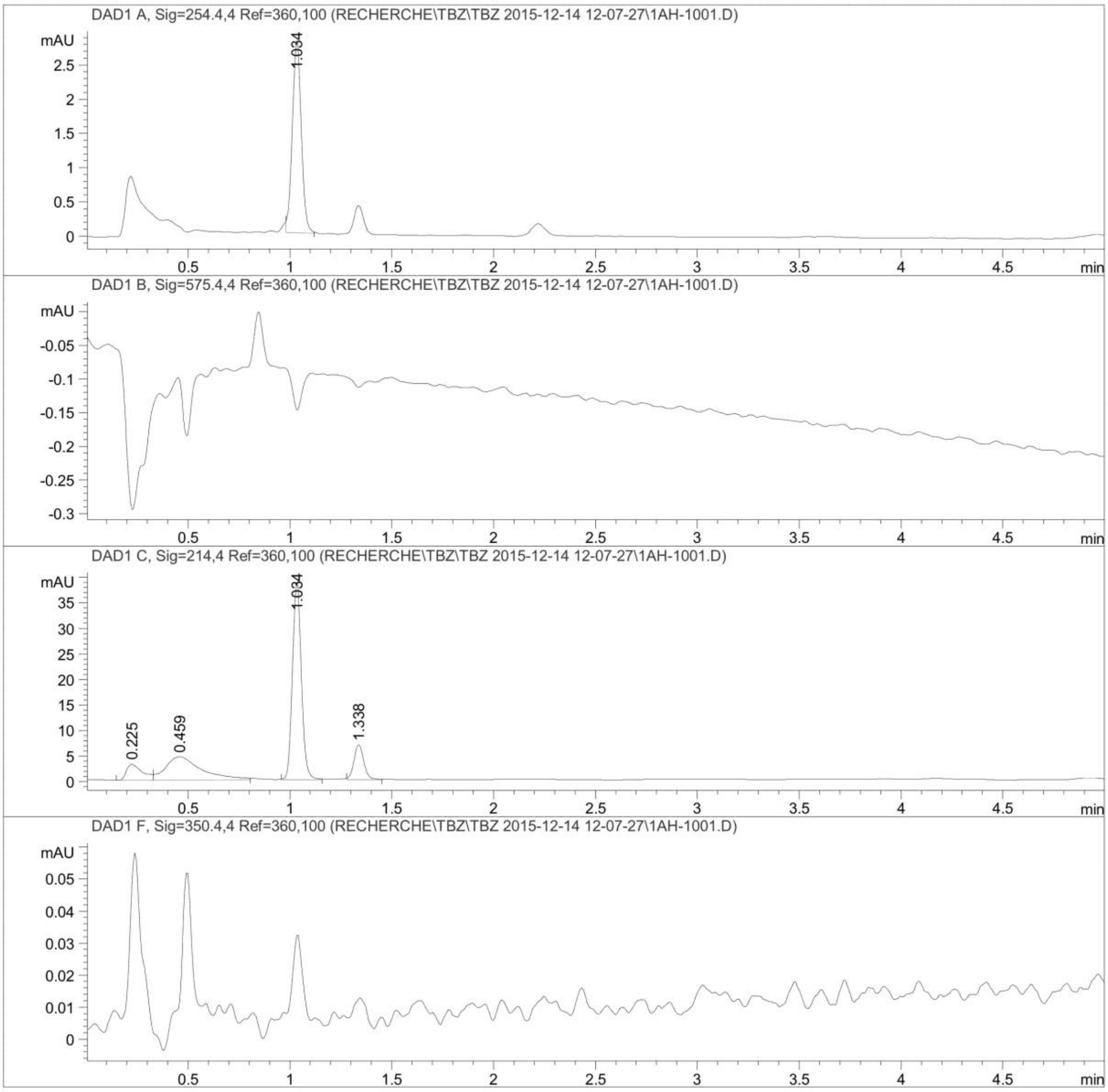

Data File C:\CHEM32\1\DATA\RECHERCHE\TBZ\TBZ 2015-12-14 12-07-27\1AH-1001.D
Sample Name: F3-stab3mois-1

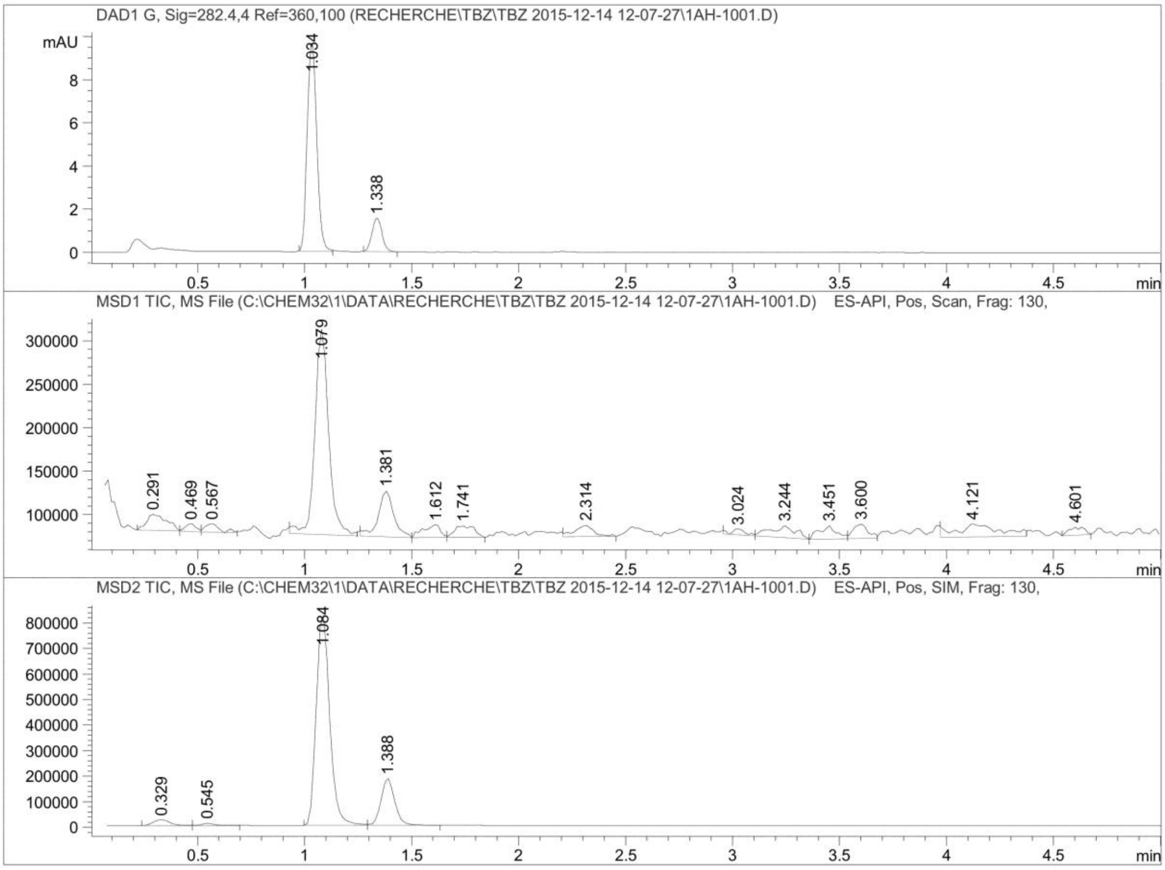


```
=====================================================================
                         Area Percent Report
=====================================================================

Sorted By             :      Signal
Multiplier            :      1.0000
Dilution              :      1.0000
Use Multiplier & Dilution Factor with ISTDs


Signal 1: DAD1 A, Sig=254.4,4 Ref=360,100

Peak RetTime Type  Width     Area      Height     Area
  #   [min]        [min]   [mAU*s]     [mAU]        %
----|-------|----|-------|----------|----------|--------|
   1   1.034 BB    0.0482    8.73121    2.81406 100.0000

Totals :                     8.73121    2.81406
```

Signal 2: DAD1 B, Sig=575.4,4 Ref=360,100

Signal 3: DAD1 C, Sig=214,4 Ref=360,100

Peak #	RetTime [min]	Type	Width [min]	Area [mAU*s]	Height [mAU]	Area %
1	0.225	BV	0.0833	16.97180	3.12622	7.8490
2	0.459	VB	0.1770	55.90657	4.56166	25.8554
3	1.034	BB	0.0481	120.13395	38.85295	55.5589
4	1.338	BB	0.0534	23.21576	6.86830	10.7367

Totals : 216.22808 53.40913

Signal 4: DAD1 F, Sig=350.4,4 Ref=360,100

Signal 5: DAD1 G, Sig=282.4,4 Ref=360,100

Peak #	RetTime [min]	Type	Width [min]	Area [mAU*s]	Height [mAU]	Area %
1	1.034	BB	0.0478	29.82697	9.72469	85.5288
2	1.338	BB	0.0518	5.04662	1.55790	14.4712

Totals : 34.87359 11.28259

Signal 6: MSD1 TIC, MS File

Peak #	RetTime [min]	Type	Width [min]	Area	Height	Area %
1	0.291	BV	0.0867	1.24104e5	1.86626e4	5.3565
2	0.469	VV	0.0483	3.09673e4	9214.93457	1.3366
3	0.567	VB	0.0722	4.38191e4	9464.25586	1.8913
4	1.079	BB	0.0661	1.06097e6	2.35907e5	45.7932
5	1.381	BV	0.0718	2.63012e5	5.26805e4	11.3521
6	1.612	VV	0.0923	8.14585e4	1.47013e4	3.5159
7	1.741	VV	0.1058	8.32286e4	1.31069e4	3.5923
8	2.314	BV	0.0798	7.76252e4	1.27976e4	3.3504
9	3.024	BV	0.0648	3.08148e4	7025.07031	1.3300
10	3.244	VV	0.1382	1.11826e5	1.40874e4	4.8266
11	3.451	VV	0.0728	8.50824e4	1.55659e4	3.6723
12	3.600	VV	0.0781	8.00424e4	1.68372e4	3.4548
13	4.121	BB	0.1645	1.99382e5	1.54109e4	8.6057
14	4.601	VV	0.0743	4.45367e4	9249.70898	1.9223

Totals : 2.31686e6 4.44711e5

Signal 7: MSD2 TIC, MS File

Peak #	RetTime [min]	Type	Width [min]	Area	Height	Area %
1	0.329	BV	0.0792	1.19141e5	2.27470e4	2.5796
2	0.545	VB	0.0640	3.42927e4	7940.96533	0.7425
3	1.084	BV	0.0692	3.63385e6	8.30728e5	78.6797
4	1.388	VB	0.0708	8.31254e5	1.84288e5	17.9982

Totals : 4.61854e6 1.04570e6

==

*** End of Report ***

Stability of F4 sample in LC-MS at 3 months

Data File C:\CHEM32\1\DATA\RECHERCHE\TBZ\TBZ 2015-12-14 12-07-27\1BB-1401.D
Sample Name: F4-stab3mois-1

===

Acq. Operator : LE-ZSL Seq. Line : 14
Acq. Instrument : LCMS Location : P1-B-02
Injection Date : 12/14/2015 1:54:52 PM Inj : 1
Inj Volume : 0.100 µl
Acq. Method : C:\CHEM32\1\DATA\RECHERCHE\TBZ\TBZ 2015-12-14 12-07-27\TBZISO_45ACN_55TP4.
Last changed : 12/14/2015 11:04:54 AM by LE-ZSL
Analysis Method : C:\CHEM32\1\DATA\RECHERCHE\TBZ\TBZ 2015-12-14 12-07-27\1BB-1401.D\DA.M (TBZISO_45ACN_55TP4.5.M, From Data File)
Last changed : 12/14/2015 11:04:54 AM by LE-ZSL

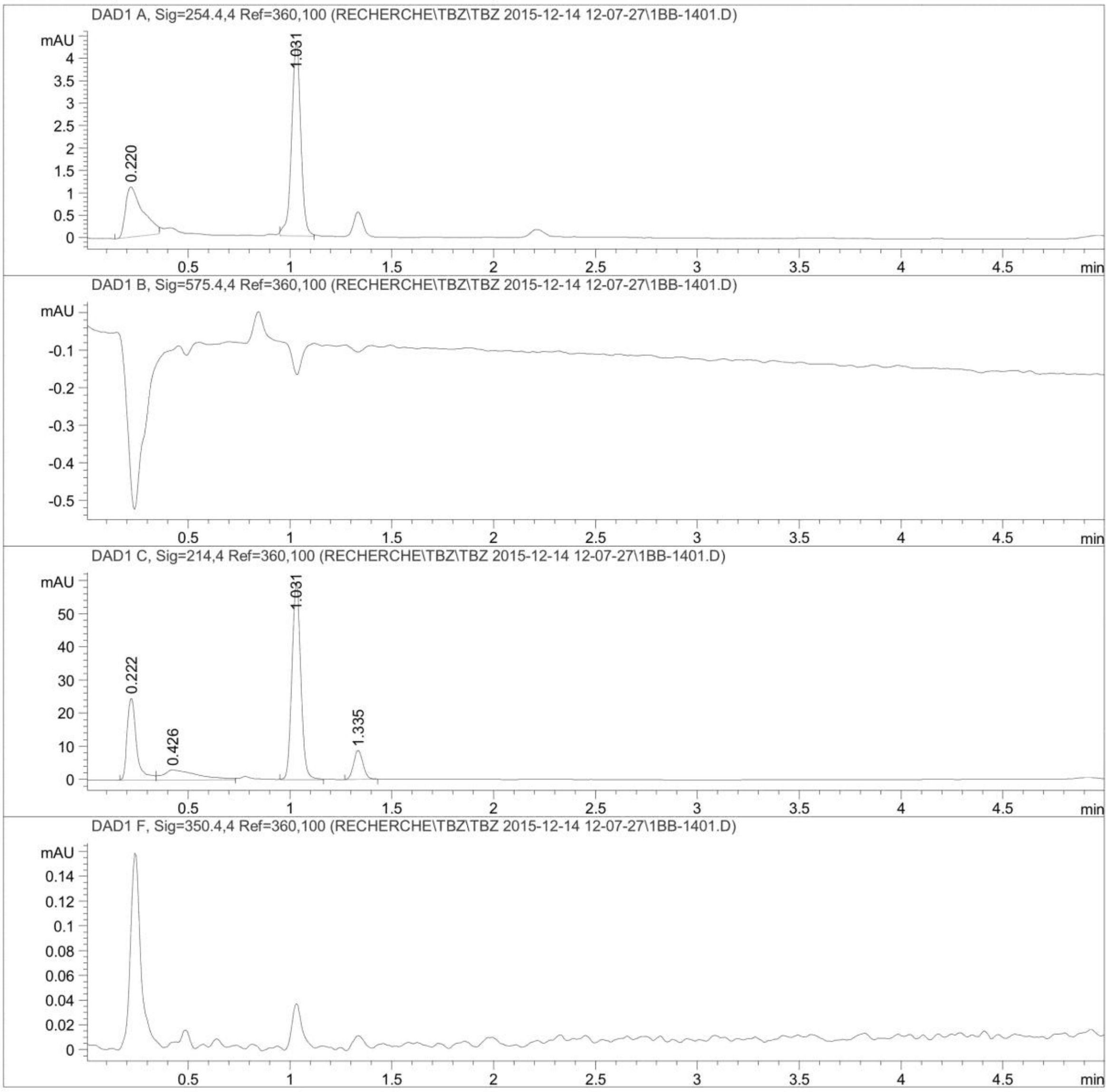

Data File C:\CHEM32\1\DATA\RECHERCHE\TBZ\TBZ 2015-12-14 12-07-27\1BB-1401.D
Sample Name: F4-stab3mois-1

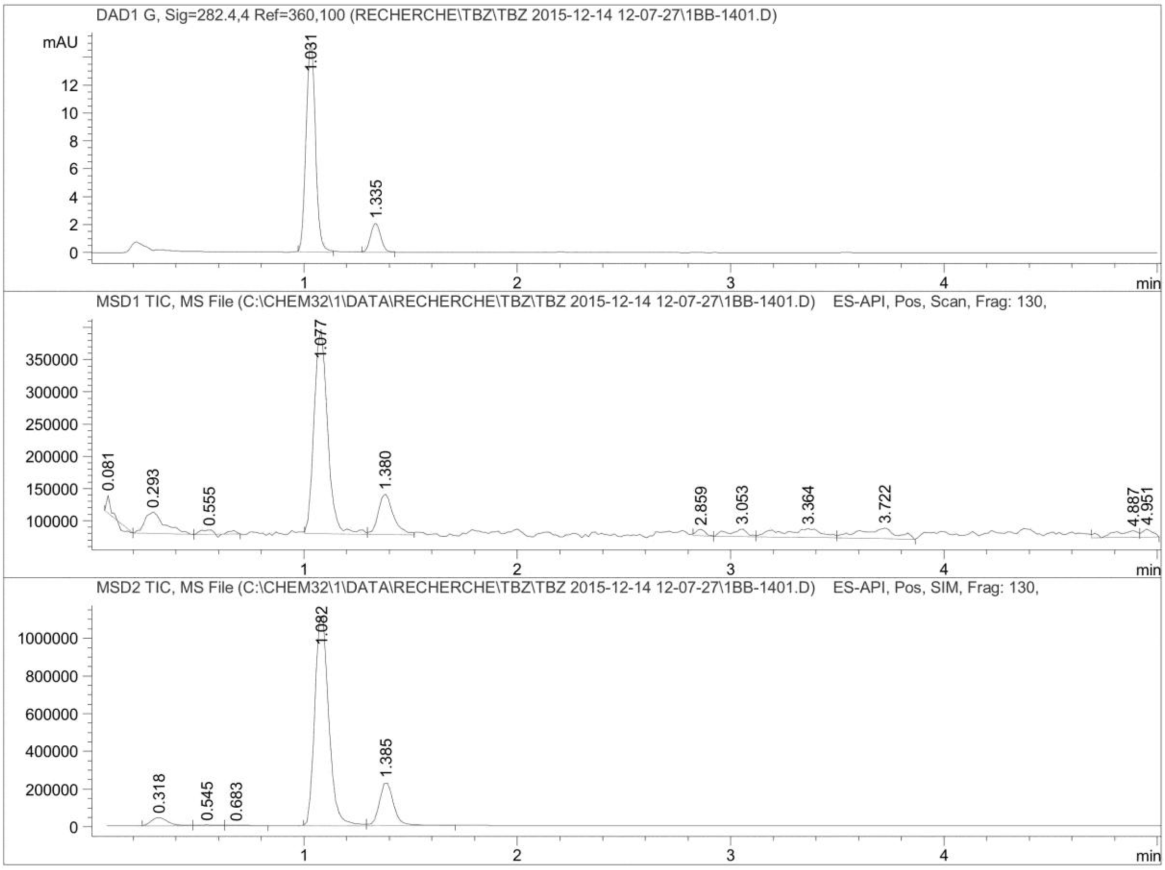

===

Area Percent Report

===

Sorted By : Signal
Multiplier : 1.0000
Dilution : 1.0000
Use Multiplier & Dilution Factor with ISTDs

Signal 1: DAD1 A, Sig=254.4,4 Ref=360,100

Peak #	RetTime [min]	Type	Width [min]	Area [mAU*s]	Height [mAU]	Area %
1	0.220	BB	0.0855	6.54668	1.13072	32.3244
2	1.031	BB	0.0488	13.70637	4.34463	67.6756

Totals : 20.25305 5.47535

Signal 2: DAD1 B, Sig=575.4,4 Ref=360,100

Signal 3: DAD1 C, Sig=214,4 Ref=360,100

Peak #	RetTime [min]	Type	Width [min]	Area [mAU*s]	Height [mAU]	Area %
1	0.222	BV	0.0492	79.51682	24.91737	24.3574
2	0.426	VB	0.1518	35.66554	3.01968	10.9250
3	1.031	BB	0.0478	182.70418	59.57926	55.9655
4	1.335	BB	0.0499	28.57217	8.78575	8.7522
Totals :				326.45872	96.30206	

Signal 4: DAD1 F, Sig=350.4,4 Ref=360,100

Signal 5: DAD1 G, Sig=282.4,4 Ref=360,100

Peak #	RetTime [min]	Type	Width [min]	Area [mAU*s]	Height [mAU]	Area %
1	1.031	BB	0.0496	45.82727	14.99925	87.1190
2	1.335	BB	0.0502	6.77583	2.07189	12.8810
Totals :				52.60310	17.07113	

Signal 6: MSD1 TIC, MS File

Peak #	RetTime [min]	Type	Width [min]	Area	Height	Area %
1	0.081	BV	8.67e-3	1.45589e4	2.79756e4	0.5610
2	0.293	VV	0.0990	2.17654e5	3.32490e4	8.3868
3	0.555	VB	0.0926	3.91305e4	7029.46631	1.5078
4	1.077	BV	0.0697	1.40717e6	3.18204e5	54.2221
5	1.380	VB	0.0761	3.11909e5	6.27638e4	12.0187
6	2.859	BV	0.0471	2.77530e4	9670.92480	1.0694
7	3.053	VV	0.0753	7.08412e4	1.24750e4	2.7297
8	3.364	VV	0.2163	1.83622e5	1.40138e4	7.0755
9	3.722	VV	0.1556	1.94876e5	1.65368e4	7.5091
10	4.887	BV	0.1002	8.47594e4	1.08524e4	3.2660
11	4.951	VBA	0.0566	4.29214e4	1.26308e4	1.6539
Totals :				2.59520e6	5.25401e5	

Signal 7: MSD2 TIC, MS File

Peak #	RetTime [min]	Type	Width [min]	Area	Height	Area %
1	0.318	BV	0.0834	2.23459e5	4.29086e4	3.5171
2	0.545	VV	0.0718	1.67076e4	3351.51831	0.2630
3	0.683	VB	0.0891	9214.29980	1515.01270	0.1450
4	1.082	BV	0.0696	5.04864e6	1.14342e6	79.4626
5	1.385	VB	0.0714	1.05546e6	2.31041e5	16.6123
Totals :				6.35348e6	1.42223e6	

===

*** End of Report ***

Stability of F1 sample in LC-MS at 6 months

Data File C:\CHEM32\1\DATA\RECHERCHE\TBZ\TBZ4 2016-05-25 10-55-35\1AC-0301.D
Sample Name: F1-STAB6M-1

===

```
Acq. Operator   : LE                              Seq. Line :   3
Acq. Instrument : LCMS                             Location : P1-A-03
Injection Date  : 5/25/2016 11:15:33 AM                 Inj :   1
                                                 Inj Volume : 0.100 µl
Acq. Method     : C:\CHEM32\1\DATA\TBZ4 2016-05-25 10-55-35\TBZISO_40ACN_60TP4.
Last changed    : 5/25/2016 10:35:48 AM by LT
Analysis Method : C:\CHEM32\1\DATA\RECHERCHE\TBZ\TBZ4 2016-05-25 10-55-35\1AC-0301.D\DA.M (
                  TBZISO_40ACN_60TP4.5.M, From Data File)
Last changed    : 5/25/2016 10:35:48 AM by LT
```

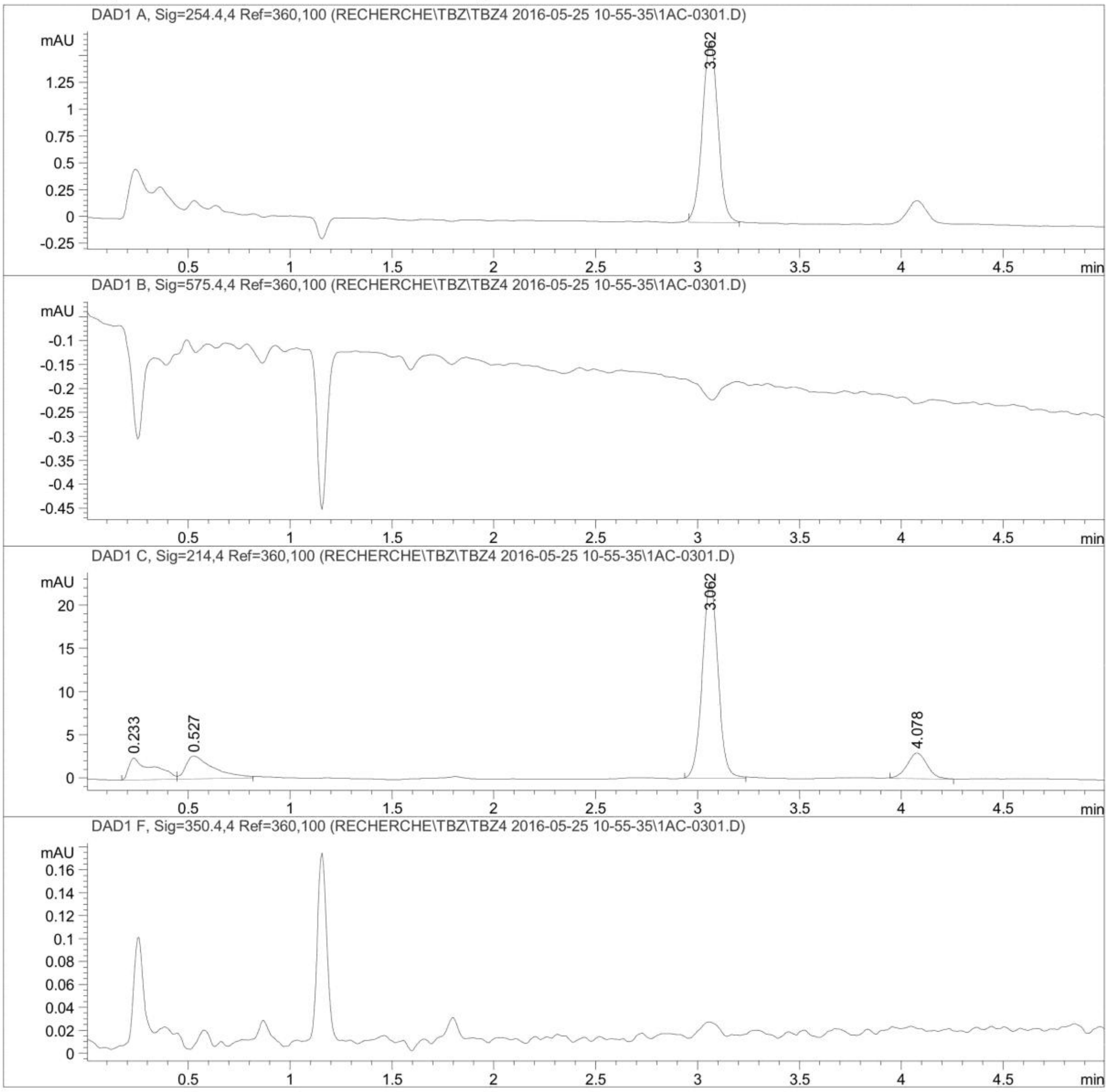

LCMS 1/4/2019 4:51:54 PM LT Page 1 of 4

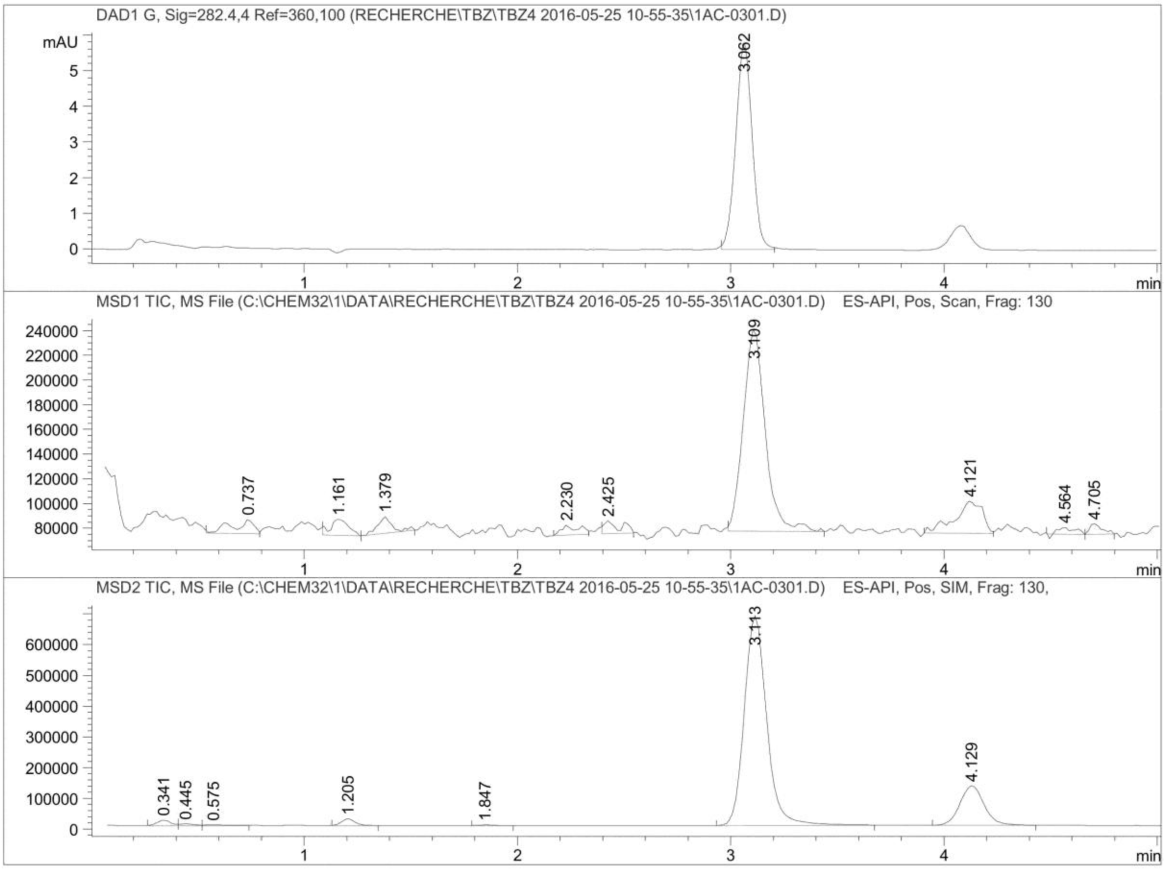

===

Area Percent Report

===

Sorted By : Signal
Multiplier : 1.0000
Dilution : 1.0000
Use Multiplier & Dilution Factor with ISTDs

Signal 1: DAD1 A, Sig=254.4,4 Ref=360,100

Peak #	RetTime [min]	Type	Width [min]	Area [mAU*s]	Height [mAU]	Area %
1	3.062	BB	0.0819	8.99476	1.69473	100.0000

Totals : 8.99476 1.69473

Data File C:\CHEM32\1\DATA\RECHERCHE\TBZ\TBZ4 2016-05-25 10-55-35\1AC-0301.D
Sample Name: F1-STAB6M-1

Peak #	RetTime [min]	Type	Width [min]	Area	Height	Area %
1	0.341	BV	0.0685	7.59641e4	1.76041e4	1.2186
2	0.445	VV	0.0748	2.36064e4	5256.76514	0.3787
3	0.575	VB	0.0695	1.38632e4	2895.68896	0.2224
4	1.205	BB	0.0691	9.46931e4	2.16694e4	1.5191
5	1.847	BB	0.0753	1.10315e4	2251.46338	0.1770
6	3.113	BB	0.1126	4.99892e6	6.82914e5	80.1940
7	4.129	BB	0.1236	1.01545e6	1.28951e5	16.2902
Totals :				6.23353e6	8.61542e5	

==

*** End of Report ***

Signal 2: DAD1 B, Sig=575.4,4 Ref=360,100

Signal 3: DAD1 C, Sig=214,4 Ref=360,100

Peak #	RetTime [min]	Type	Width [min]	Area [mAU*s]	Height [mAU]	Area %
1	0.233	BV	0.1088	20.89197	2.51770	11.1862
2	0.527	VB	0.1318	24.44398	2.64577	13.0880
3	3.062	BB	0.0824	121.36701	22.67712	64.9834
4	4.078	BB	0.1053	20.06318	2.95292	10.7424

Totals : 186.76614 30.79350

Signal 4: DAD1 F, Sig=350.4,4 Ref=360,100

Signal 5: DAD1 G, Sig=282.4,4 Ref=360,100

Peak #	RetTime [min]	Type	Width [min]	Area [mAU*s]	Height [mAU]	Area %
1	3.062	BB	0.0822	30.94361	5.80367	100.0000

Totals : 30.94361 5.80367

Signal 6: MSD1 TIC, MS File

Peak #	RetTime [min]	Type	Width [min]	Area	Height	Area %
1	0.737	BV	0.1275	7.75104e4	1.16507e4	4.3448
2	1.161	BV	0.0905	7.58042e4	1.30437e4	4.2492
3	1.379	VB	0.0637	6.31274e4	1.34798e4	3.5386
4	2.230	BB	0.0790	4.48119e4	7990.03174	2.5119
5	2.425	BB	0.0822	5.57451e4	1.04971e4	3.1248
6	3.109	BB	0.1104	1.17010e6	1.64071e5	65.5899
7	4.121	BV	0.1187	2.33770e5	2.59474e4	13.1040
8	4.564	BV	0.0682	2.78029e4	5489.31152	1.5585
9	4.705	VB	0.0607	3.52920e4	8733.84473	1.9783

Totals : 1.78397e6 2.60903e5

Signal 7: MSD2 TIC, MS File

Stability of F2 sample in LC-MS at 3 months

Data File C:\CHEM32\1\DATA\RECHERCHE\TBZ\TBZ4 2016-05-25 10-55-35\1AF-0701.D
Sample Name: F2-STAB6M-1

===

Acq. Operator : LE Seq. Line : 7
Acq. Instrument : LCMS Location : P1-A-06
Injection Date : 5/25/2016 11:40:31 AM Inj : 1
Inj Volume : 0.100 µl
Acq. Method : C:\CHEM32\1\DATA\TBZ4 2016-05-25 10-55-35\TBZISO_40ACN_60TP4.
Last changed : 5/25/2016 10:35:48 AM by LT
Analysis Method : C:\CHEM32\1\DATA\RECHERCHE\TBZ\TBZ4 2016-05-25 10-55-35\1AF-0701.D\DA.M (
TBZISO_40ACN_60TP4.5.M, From Data File)
Last changed : 5/25/2016 10:35:48 AM by LT

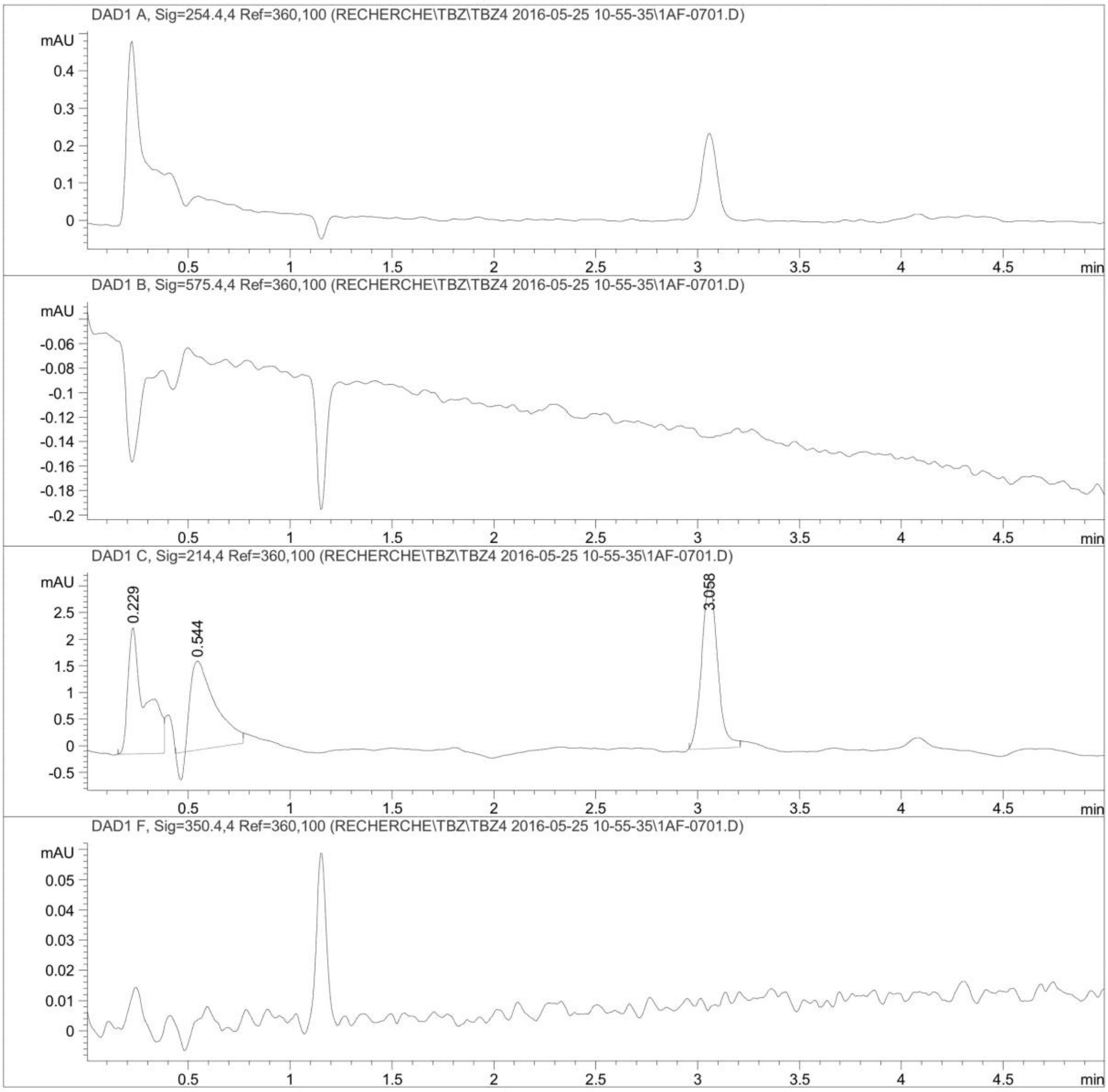

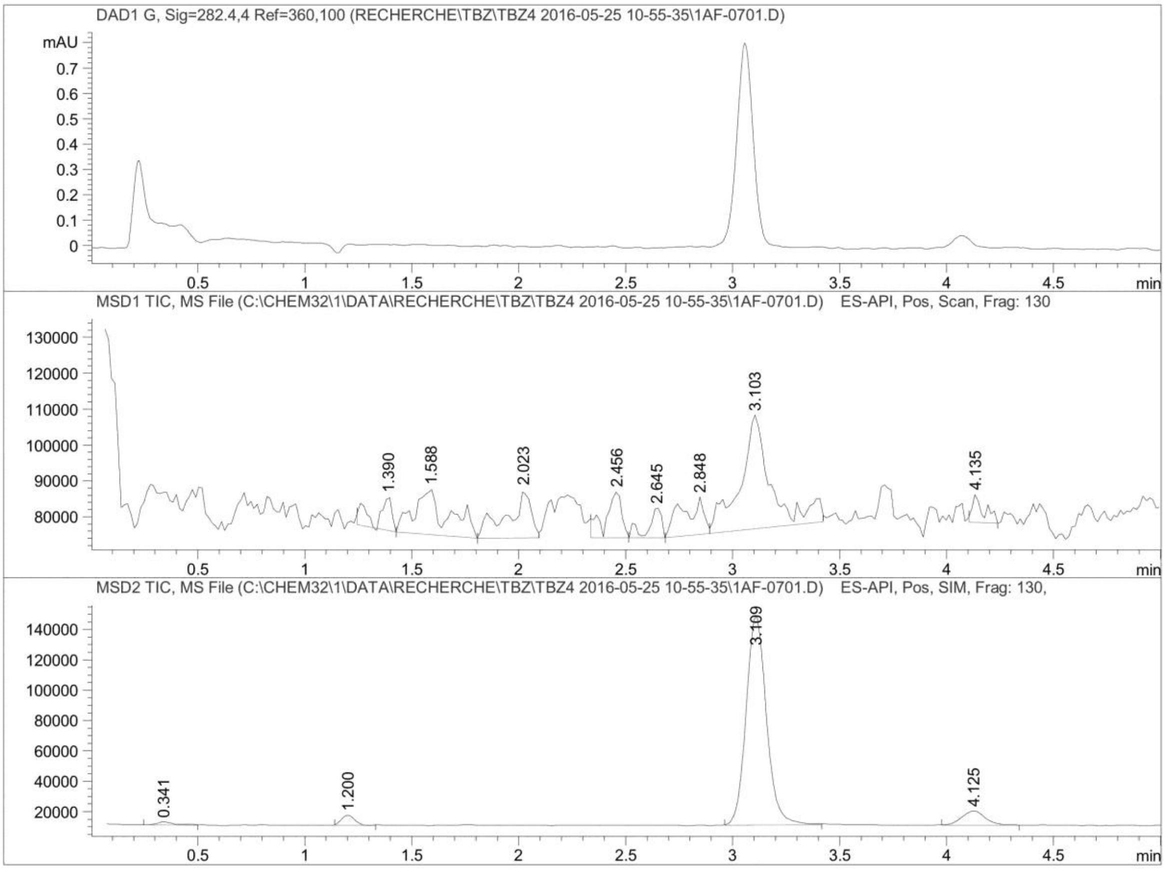

===
Area Percent Report
===

Sorted By : Signal
Multiplier : 1.0000
Dilution : 1.0000
Use Multiplier & Dilution Factor with ISTDs

Signal 1: DAD1 A, Sig=254.4,4 Ref=360,100

Signal 2: DAD1 B, Sig=575.4,4 Ref=360,100

Signal 3: DAD1 C, Sig=214,4 Ref=360,100

Peak #	RetTime [min]	Type	Width [min]	Area [mAU*s]	Height [mAU]	Area %
1	0.229	BB	0.0836	14.18466	2.37852	31.9992
2	0.544	BB	0.1204	13.24462	1.67256	29.8786
3	3.058	BB	0.0831	16.89888	3.12323	38.1222

Data File C:\CHEM32\1\DATA\RECHERCHE\TBZ\TBZ4 2016-05-25 10-55-35\1AF-0701.D
Sample Name: F2-STAB6M-1

Peak #	RetTime [min]	Type	Width [min]	Area [mAU*s]	Height [mAU]	Area %
Totals :				44.32815	7.17431	

Signal 4: DAD1 F, Sig=350.4,4 Ref=360,100

Signal 5: DAD1 G, Sig=282.4,4 Ref=360,100

Signal 6: MSD1 TIC, MS File

Peak #	RetTime [min]	Type	Width [min]	Area	Height	Area %
1	1.390	BV	0.0653	4.58022e4	9503.51367	5.6916
2	1.588	VV	0.1438	1.36560e5	1.26781e4	16.9696
3	2.023	VV	0.1291	1.02132e5	1.34896e4	12.6914
4	2.456	BV	0.0755	6.81461e4	1.28447e4	8.4681
5	2.645	VV	0.0628	3.47071e4	8448.28906	4.3129
6	2.848	VV	0.1224	7.28225e4	1.04182e4	9.0493
7	3.103	VB	0.1344	3.15452e5	3.16659e4	39.1995
8	4.135	BB	0.0620	2.91124e4	7822.20752	3.6176
Totals :				8.04735e5	1.06871e5	

Signal 7: MSD2 TIC, MS File

Peak #	RetTime [min]	Type	Width [min]	Area	Height	Area %
1	0.341	BB	0.0839	1.17915e4	2090.77100	1.1533
2	1.200	BB	0.0679	2.85292e4	6683.57910	2.7905
3	3.109	BB	0.1037	9.11840e5	1.39093e5	89.1875
4	4.125	BB	0.1196	7.02245e4	9328.27051	6.8687
Totals :				1.02238e6	1.57196e5	

==

*** End of Report ***

Stability of F3 sample in LC-MS at 6 months

```
Data File C:\CHEM32\1\DATA\RECHERCHE\TBZ\TBZ4 2016-05-25 10-55-35\1AI-1101.D
Sample Name: F3-STAB6M-1

    =====================================================================
    Acq. Operator   : LE                             Seq. Line :  11
    Acq. Instrument : LCMS                            Location : P1-A-09
    Injection Date  : 5/25/2016 12:05:34 PM                Inj :   1
                                                    Inj Volume : 0.100 µl
    Acq. Method     : C:\CHEM32\1\DATA\TBZ4 2016-05-25 10-55-35\TBZISO_40ACN_60TP4.
    Last changed    : 5/25/2016 10:35:48 AM by LT
    Analysis Method : C:\CHEM32\1\DATA\RECHERCHE\TBZ\TBZ4 2016-05-25 10-55-35\1AI-1101.D\DA.M (
                      TBZISO_40ACN_60TP4.5.M, From Data File)
    Last changed    : 5/25/2016 10:35:48 AM by LT
```

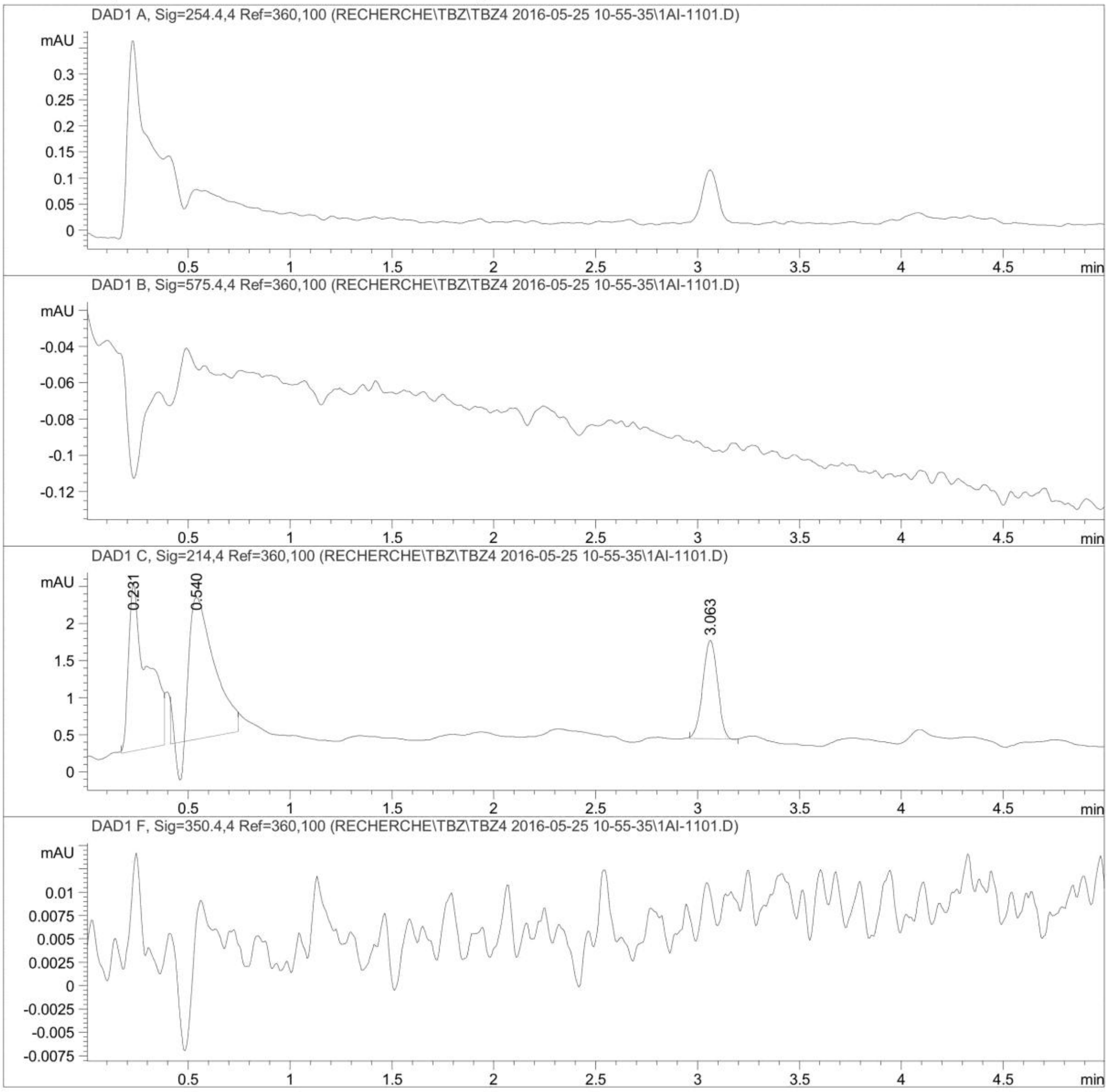

Data File C:\CHEM32\1\DATA\RECHERCHE\TBZ\TBZ4 2016-05-25 10-55-35\1AI-1101.D
Sample Name: F3-STAB6M-1

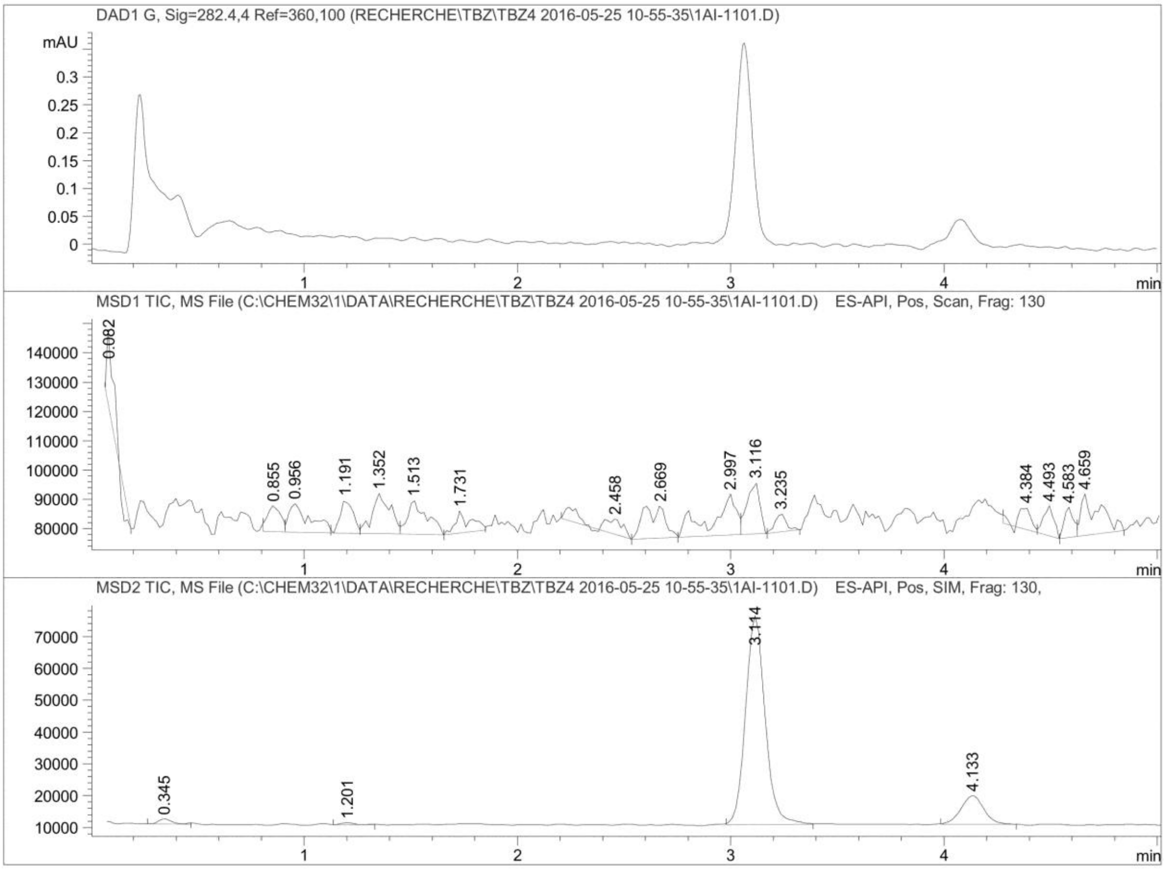

===

Area Percent Report

===

Sorted By : Signal
Multiplier : 1.0000
Dilution : 1.0000
Use Multiplier & Dilution Factor with ISTDs

Signal 1: DAD1 A, Sig=254.4,4 Ref=360,100

Signal 2: DAD1 B, Sig=575.4,4 Ref=360,100

Signal 3: DAD1 C, Sig=214,4 Ref=360,100

Peak #	RetTime [min]	Type	Width [min]	Area [mAU*s]	Height [mAU]	Area %
1	0.231	BB	0.0860	14.43095	2.27389	39.4070
2	0.540	BB	0.1227	15.54989	1.91599	42.4625
3	3.063	BB	0.0782	6.63947	1.33123	18.1306

Peak #	RetTime [min]	Type	Width [min]	Area [mAU*s]	Height [mAU]	Area %
Totals :				36.62031	5.52111	

Signal 4: DAD1 F, Sig=350.4,4 Ref=360,100

Signal 5: DAD1 G, Sig=282.4,4 Ref=360,100

Signal 6: MSD1 TIC, MS File

Peak #	RetTime [min]	Type	Width [min]	Area	Height	Area %
1	0.082	BB	0.0259	4.05200e4	2.60602e4	4.5275
2	0.855	BV	0.0722	3.77943e4	8804.27734	4.2230
3	0.956	VB	0.1021	6.55575e4	9643.90918	7.3251
4	1.191	BV	0.0755	4.68967e4	1.13223e4	5.2400
5	1.352	VV	0.0824	8.11455e4	1.37654e4	9.0669
6	1.513	VV	0.0819	7.28132e4	1.16698e4	8.1359
7	1.731	VB	0.0818	3.70315e4	7541.32422	4.1378
8	2.458	BV	0.1339	4.88564e4	5341.93652	5.4590
9	2.669	VV	0.1142	7.84644e4	1.10947e4	8.7673
10	2.997	VV	0.1000	1.09383e5	1.40290e4	12.2220
11	3.116	VV	0.0671	7.37081e4	1.75507e4	8.2358
12	3.235	VV	0.0606	2.21801e4	6091.88623	2.4783
13	4.384	BV	0.0646	3.03107e4	7606.04785	3.3868
14	4.493	VV	0.0524	3.43287e4	1.03097e4	3.8358
15	4.583	VV	0.0440	3.09901e4	1.03569e4	3.4627
16	4.659	VB	0.0788	8.49867e4	1.42210e4	9.4961
Totals :				8.94967e5	1.85409e5	

Signal 7: MSD2 TIC, MS File

Peak #	RetTime [min]	Type	Width [min]	Area	Height	Area %
1	0.345	BB	0.0729	7335.42432	1562.69519	1.4773
2	1.201	BB	0.0676	2819.18579	664.38837	0.5678
3	3.114	BB	0.0972	4.19031e5	6.56019e4	84.3891
4	4.133	BB	0.1141	6.73605e4	9042.93164	13.5658
Totals :				4.96546e5	7.68719e4	

===
*** End of Report ***

Stability of F4 sample in LC-MS at 6 months

```
Data File C:\CHEM32\1\DATA\RECHERCHE\TBZ\TBZ4 2016-05-25 10-55-35\1BC-1501.D
Sample Name: F4-STAB6M-1

    =====================================================================
    Acq. Operator   : LE                            Seq. Line :  15
    Acq. Instrument : LCMS                           Location : P1-B-03
    Injection Date  : 5/25/2016 12:30:40 PM               Inj :   1
                                                   Inj Volume : 0.100 µl
    Acq. Method     : C:\CHEM32\1\DATA\TBZ4 2016-05-25 10-55-35\TBZISO_40ACN_60TP4.
    Last changed    : 5/25/2016 10:35:48 AM by LT
    Analysis Method : C:\CHEM32\1\DATA\RECHERCHE\TBZ\TBZ4 2016-05-25 10-55-35\1BC-1501.D\DA.M (
                      TBZISO_40ACN_60TP4.5.M, From Data File)
    Last changed    : 5/25/2016 10:35:48 AM by LT
```

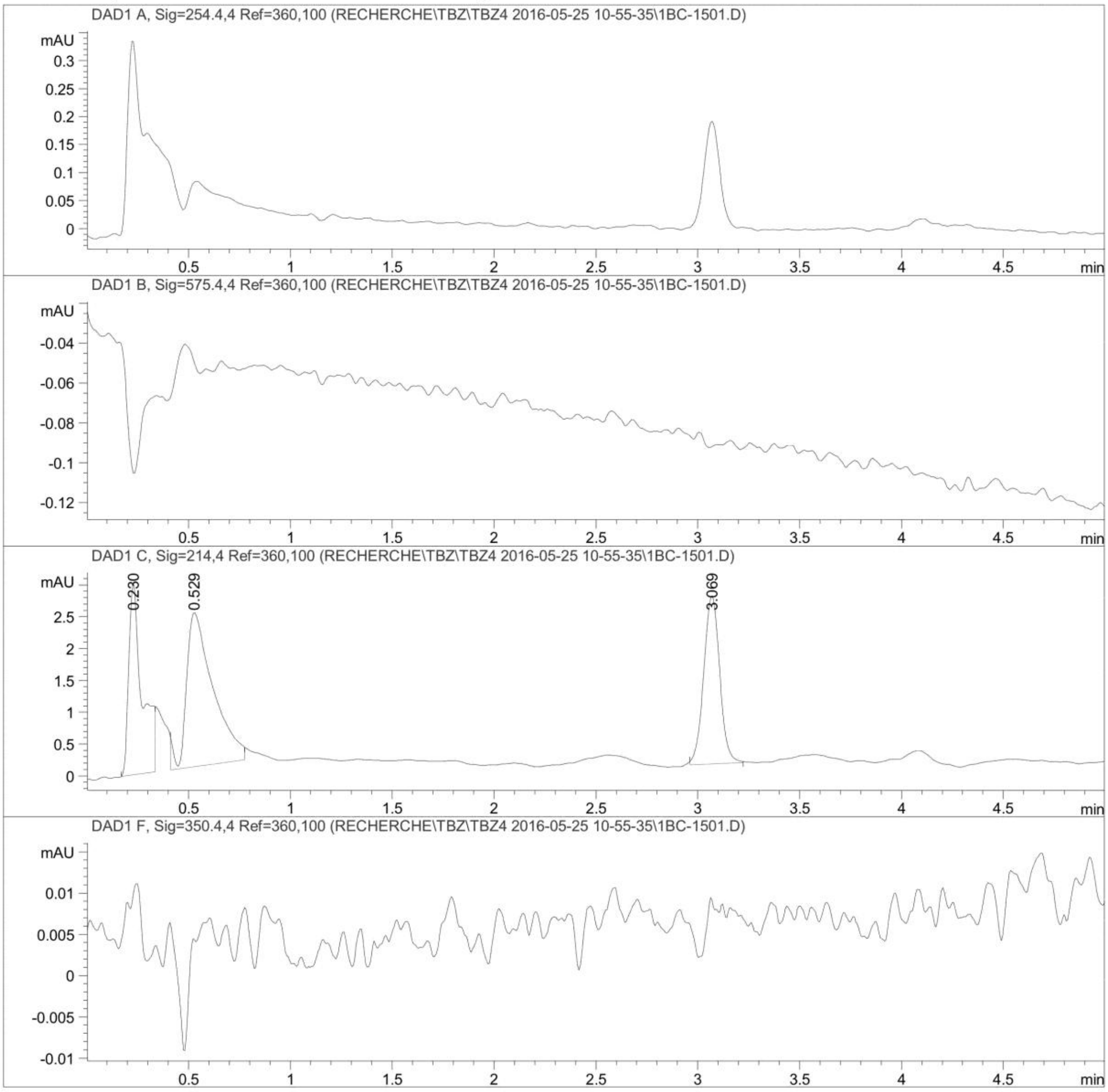

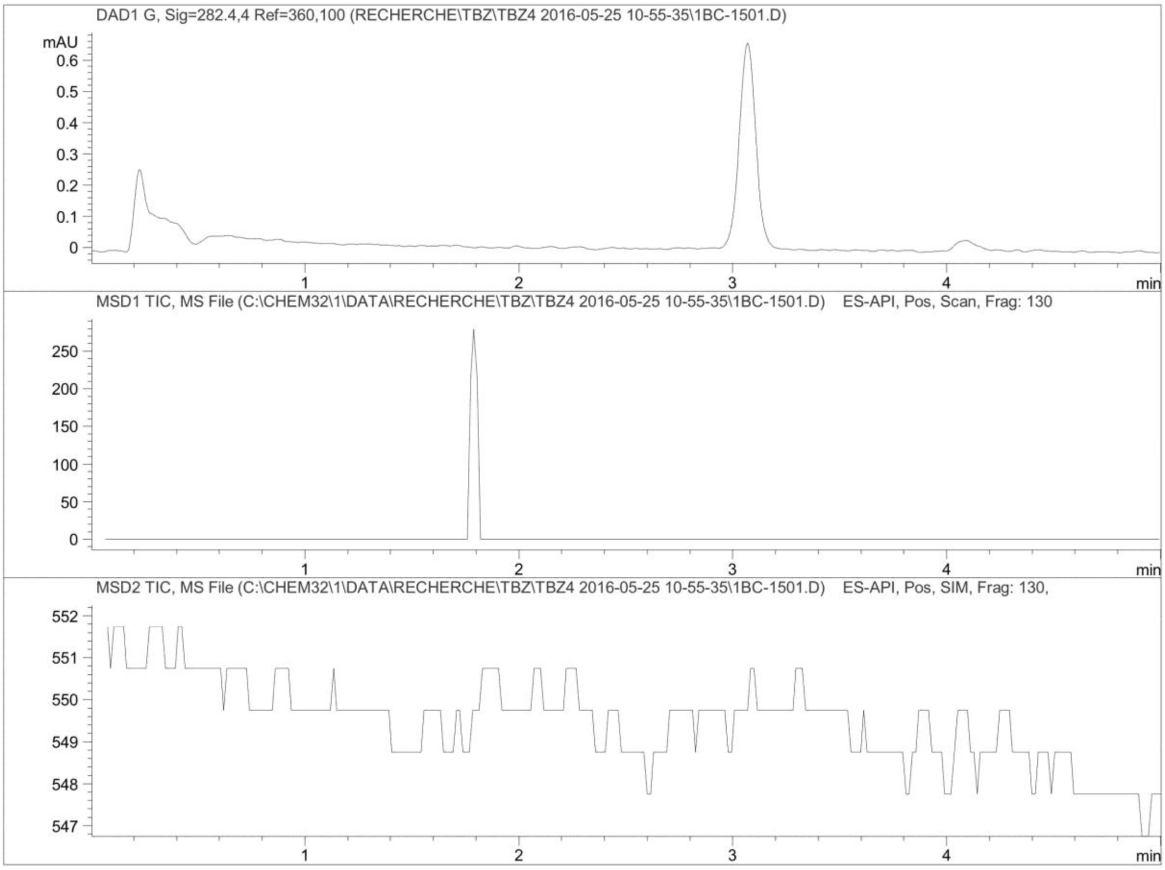

===

Area Percent Report

===

Sorted By : Signal
Multiplier : 1.0000
Dilution : 1.0000
Use Multiplier & Dilution Factor with ISTDs

Signal 1: DAD1 A, Sig=254.4,4 Ref=360,100

Signal 2: DAD1 B, Sig=575.4,4 Ref=360,100

Signal 3: DAD1 C, Sig=214,4 Ref=360,100

Peak #	RetTime [min]	Type	Width [min]	Area [mAU*s]	Height [mAU]	Area %
1	0.230	BB	0.0645	13.61779	3.02719	27.2859
2	0.529	BB	0.1296	21.92267	2.42226	43.9264
3	3.069	BB	0.0834	14.36729	2.64560	28.7877

```
Data File C:\CHEM32\1\DATA\RECHERCHE\TBZ\TBZ4 2016-05-25 10-55-35\1BC-1501.D
Sample Name: F4-STAB6M-1

    Peak RetTime Type  Width     Area      Height     Area
      #   [min]        [min]   [mAU*s]     [mAU]        %
    ----|-------|----|-------|----------|----------|--------|
    Totals :                   49.90774    8.09505



    Signal 4: DAD1 F, Sig=350.4,4 Ref=360,100


    Signal 5: DAD1 G, Sig=282.4,4 Ref=360,100


    Signal 6: MSD1 TIC, MS File


    Signal 7: MSD2 TIC, MS File

    =====================================================================
                              *** End of Report ***
```

```
LCMS 1/4/2019 4:53:37 PM LT                                          Page   3 of 3
```

Figure S6. LC-MS data for TBZ analyses contained in ODF.

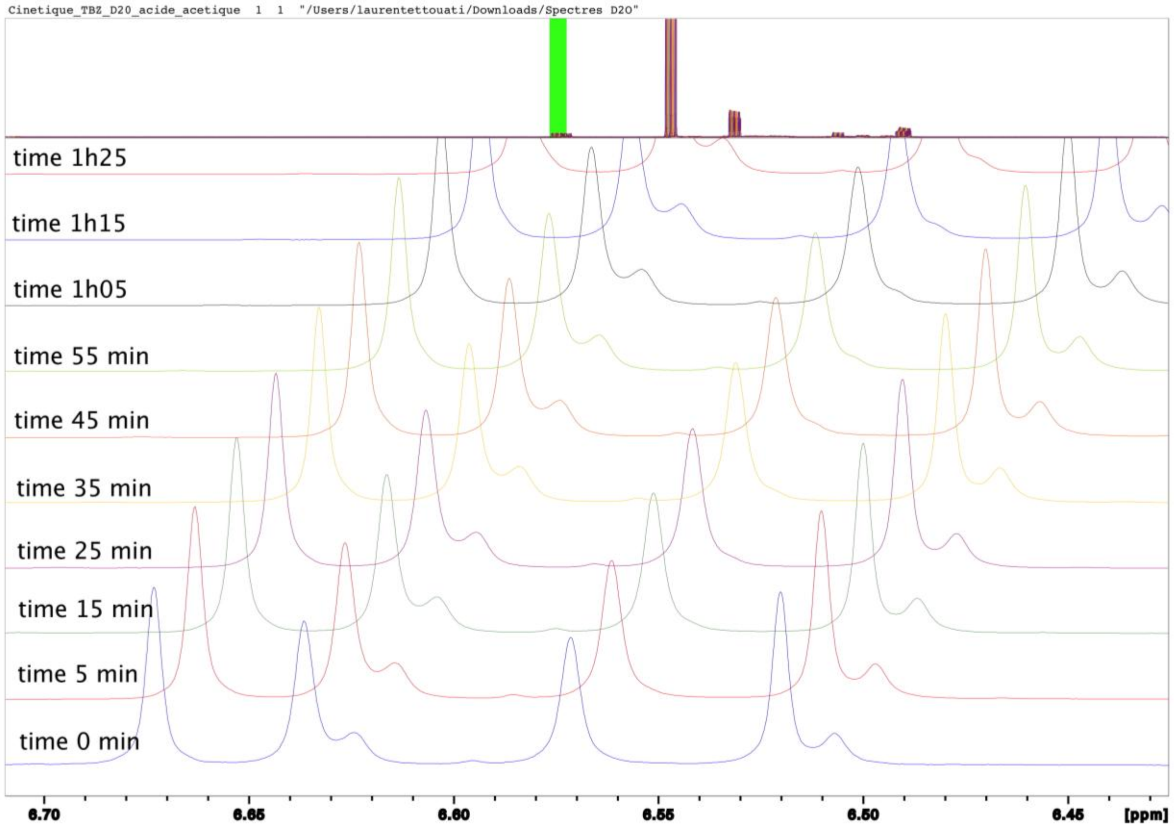

Figure S7. Kinetic experiments on TBZ in acetic acid-d4 / D_2O (10 mg TBZ plus 0.4 mL D_2O plus 4 drops of acetic acid-d4) over time at 24°C. ^{1}H NMR spectra in aromatic protons area every 10 min.

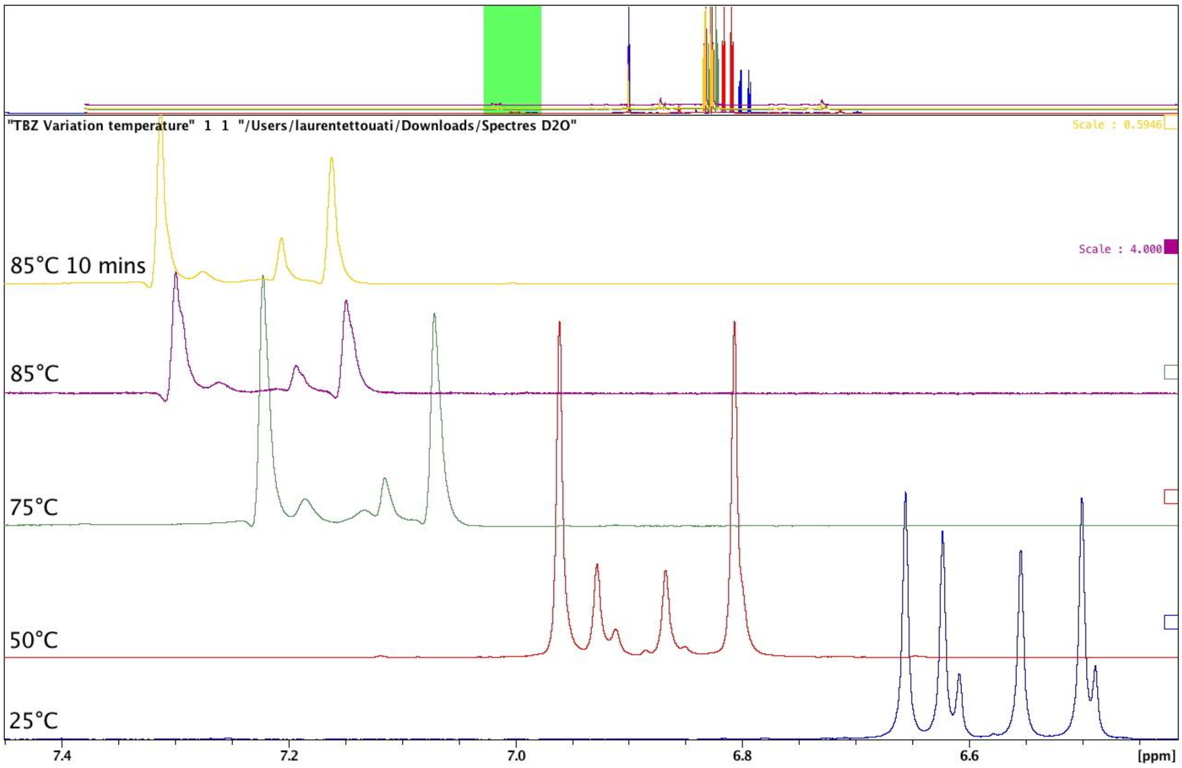

Figure S8. Kinetic experiments on TBZ in citric acid / D_2O (10 mg TBZ plus 0.4 mL D_2O plus 4 drops of citric acid 1.5 M in D_2O) at variable temperatures. ^{1}H NMR spectra in aromatic protons area with increasing temperature.
